# Detachment of secondary dendrite arm in a directionally solidified Sn-Ni peritectic alloy under deceleration growth condition

**DOI:** 10.1038/srep27682

**Published:** 2016-06-08

**Authors:** Peng Peng, Xinzhong Li, Jiangong Li, Yanqing Su, Jingjie Guo, Hengzhi Fu

**Affiliations:** 1 Institute of Materials Science and Engineering, Lanzhou University, Lanzhou, China; 2 School of Physical Science and Technology, Lanzhou University, China; 3 School of Materials Science and Engineering, Harbin Institute of Technology, China

## Abstract

In order to better understand the detachment mechanism of secondary dendrite arm during peritectic solidification, the detachment of secondary dendrite arm from the primary dendrite arms in directionally solidified Sn-36at.%Ni peritectic alloys is investigated at different deceleration rates. Extensive detachment of secondary dendrite arms from primary stem is observed below peritectic reaction temperature T_P_. And an analytical model is established to characterize the detachment process in terms of the secondary dendrite arm spacing *λ*_2_, the root radius of detached arms and the specific surface area (*S*_*V*_) of dendrites. It is found that the detachment mechanism is caused by not only curvature difference between the tips and roots of secondary branches, but also that between the thicker secondary branches and the thinner ones. Besides, this detachment process is significantly accelerated by the temperature gradient zone melting (TGZM) effect during peritectic solidification. It is demonstrated that the reaction constant (*f*) which is used to characterize the kinetics of peritectic reaction is crucial for the determination of the detachment process. The value of *f* not only changes with growth rate but also with solidification time at a given deceleration rate. In conclusion, these findings help the better understanding of the detachment mechanism.

One of the most visually prominent features of dendritic structures is the secondary side branch. The coarsening process induced by the Gibbs-Thomson effect will come about in secondary and higher order dendrite arms, if the dendrite microstructure is held in the mushy zone for a long enough period of time^1^. Many coarsening mechanisms^2^,^3^,^4^,^5^,^6^ have shown that this capillary-driven diffusional process could be characterized by secondary dendrite arm spacing (*λ*_2_). Besides, the specific surface density (specific surface or surface volume ratio) *S*_*V*_ is a more appropriate global parameter for describing coarsening microstructures in most cases because it is directly related to the interfacial free energy which is the driving force of the coarsening process. In addition to the coarsening process, the detachment of secondary arms and higher order dendrite arms might occur within the developing dendritic mushy zone^7^ if dendritic structures were held long enough either isothermally or during solidification. It has been proposed that this detachment which occurred in the mushy zone could also be a source of stray crystal^7^,^8^,^9^,^10^,^11^,^12^,^13^,^14^ during deceleration growth, thus influencing the following mechanical properties of solidification structures^15^,^16^,^17^. Especially in the superalloys where the the stray crystal formed due to the detachment of secondary dendrite arms, the stray crystal usually owns an orientation which deviates from the favorable <001> direction, this will significantly decrease the mechanical properties of the superalloys which are used for aerospace and turbine blades.

In addition, detachment can also be initiated by some other localized mechanisms that perturb the balance between the liquid and the adjacent mush network to cause a curvature-driven remelting^18^. The evidence of detachment was found by side-branch remelting^19^ during *in situ* study on detachment using transparent organic systems, which enabled the role of solute trapping and local solute/solvent diffusion in the neck regions associated with side-arm attachment^20^ to be clarified. Given these considerations, detachment of secondary dendrite arms obviously occurs as a natural part of dendrite ripening processes^18^. However, other mechanisms for initiation of local remelting have also been proposed: local pileup of solute in the mush is caused by liquid flow or velocity fluctuations at the growth front^16^,^20^ or alternatively by local internal heat sources such as recalescence from solidification of remaining interdendritic melt^17^. Among them, the detachment of secondary dendrite arm can hardly be observed during the directional solidification with a constant growth rate. Under steady state conditions, the ripening or coarsening in the mushy zone does not involve any significant detachment of side arms except for a very long period of time. However, with fluctuations in the imposed growth rate, the detachment of side arms can be more easily observed in other directional growth with varied growth conditions. Especially in directional solidification experiments with decelerating growth rate which were first carried out by Jackson *et al.*^8^. It was demonstrated that due to the deceleration of the imposed growth rate, extensive melting off dendrite arms could be observed in directionally solidified SCN-5.5wt.%H_2_O^21^ and CBr_4_-C_2_Cl_6_ samples^22^. Similar consequences were obtained in the NH_4_Cl-H_2_O system^7^.

While steady-state directional solidification is an important academic paradigm, it is not a realistic representation of the conditions prevalent during industrial casting, which normally occurs under rapidly changing growth conditions. In industrial casting, solidification normally occurs under rapidly changing growth conditions. Compared with the temperature gradient, growth velocity can be more easily changed, thus the growth velocity (*v*) is the most popular alternating parameter in solidification under non steady state growth conditions. In solidifying complex shapes like turbine blades, cross sectional area changes could lead to changes in the local growth velocity (*v*). These changes in growth velocity can lead to undesirable structures which would adversely affect the performance of the directionally solidified component.

The analyses on detachment discussed above are mainly focused on solidification with sudden decrease of growth rate, and few researches have been conducted on solidification with stepwise deceleration of growth velocity. Sudden decease of growth rate can accelerate the detachment of side arms instantaneously, but this acceleration of detachment will disappear after a sufficient long period of time. Furthermore, the detachment process which is really interesting and needs to be clarified can not be cleary observed after a sudden decrease growth condition. In the case of sudden decease of growth velocity, the detachment of side arms occurs only in the vicinity of the interface where the growth velocity decreases suddenly. As a result, the effect of decelerating growth velocity on the detachment of secondary dendrites is in a limited area whereas the detachment process will experience a longer period of time. On the contrary, gradual variation of the dendritic structures during the detachment process can be observed in the case of stepwise decreasing growth. Therefore, it is of great significance to describe the detachment of secondary dendrite arms during the solidification process with stepwise decrease of growth velocity.

Dendrite morphology has been frequently encountered in many peritectic alloys with industrial applications, such as Ti-Al^23^, Fe-Ni^24^, Pb-Bi^25^, Nd-Fe-B^26^ and Al-Ni^27^ *et al.* Previous works have demonstrated the obvious retard/acceleration of dendrite coarsening by peritectic reaction^28^/temperature gradient zone melting (TGZM) effect^27^,^29^. However, the detachment of secondary dendrite arm which is closely related to the coarsening process has never been analyzed in a peritectic system. Nor does attention has been paid to research on the detachment of secondary dendrite arm through alternately changing growth rate in peritectic systems. To better understand this detachment process during peritectic solidification, the present paper aims to characterize the detachment of secondary dendrite arm in response to the deceleration growth condition in directionally solidified Sn-36at.%Ni peritectic alloy. Although it has been confirmed that the coarsening process could be more accurately described through *S*_*V*_^28^,^29^,^30^,^31^, the detachment can not be directly observed from specific surface area *S*_*V*_. Thus both secondary dendrite arm spacing λ_2_ and *S*_*V*_ will be used to describe the detachment during coarsening process in the present work. In this work, the detachment of secondary dendrite arm from the primary dendrite stem in directionally solidified Sn-36at.%Ni peritectic alloy was investigated with an analytical model. The dependences of both λ_2_, *S*_*V*_ and root radius of detached arm *R*_*root*_ on solidification time, peritectic reaction and deceleration rate were calculated and compared with experimental results.

## Results

### Experimental results

**Figure 1:**
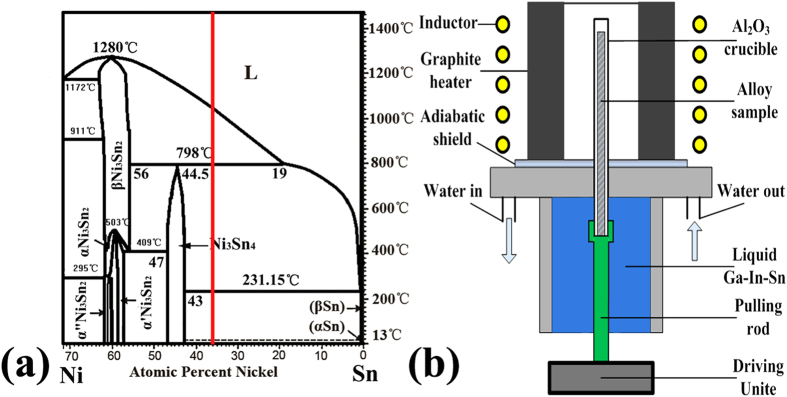
The alloy and directional solidification furnace used in this work: **(a)** relevant part of Sn-Ni binary phase diagram^32^ and **(b)** the schematic illustration of the Bridgman-type furnace used in this work.

**Figure 2:**
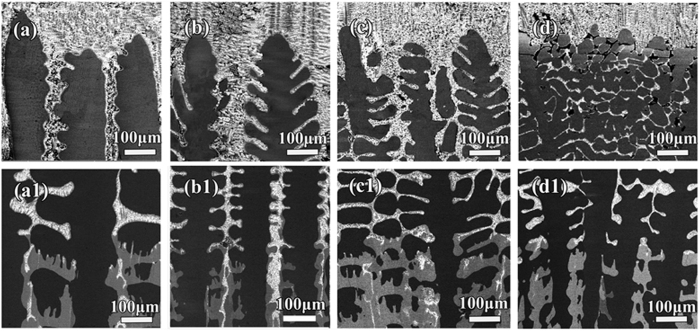
Microstructure of directionally solidified Sn-36at.%Ni peritectic alloys at the quenched solid/liquid interface (**a–d**) and peritectic interface (a1–d1) at different deceleration rates: **(a)** −2.22 × 10^−10^ m/s^2^, **(b)** −5 × 10^−10^ m/s^2^, **(c)** −8 × 10^−10^ m/s^2^ **(d)** −16 × 10^−10^ m/s^2^.

**Figure 3:**
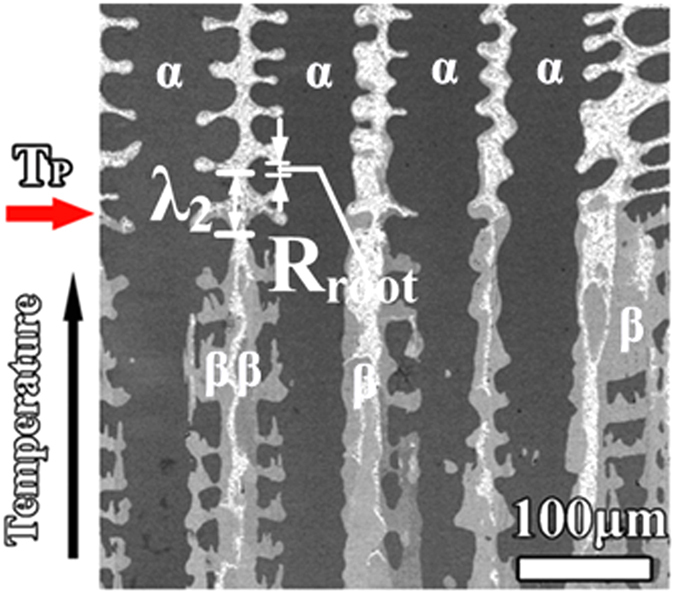
Enlarged view of the detachment process in directionally solidified Sn-36at.%Ni peritectic alloys at the deceleration rate of −5 × 10^−10^ m/s^2^.

As shown in Fig. 1a, the equilibrium solidification of Sn-36at.%Ni alloy begins^32^ at T_L_ = 1040 °C: L→Ni_3_Sn_2_; then at T_P_ = 798 °C: L+Ni_3_Sn_2_→Ni_3_Sn_4_; the remaining liquid will transform to the eutectic at T_E_ = 231.15 °C. The illustration of the Bridgman-type directional solidification furnace used in this work is shown in Fig. 1b. This furnace is consisting of a resistance furnace, a water cooled liquid metal bath filled with a liquid Ga-In-Sn alloy, and an adiabatic zone which is located between the heater and the cooler, as previously described^28^,^29^. The microstructures of directionally solidified Sn-36at.%Ni peritectic alloy at different deceleration rates (−2.22 × 10^−10^ ~ −16 × 10^−10^m/s^2^) are presented in Fig. 2. Figure 2(a–d) show the microstructures at the solid/liquid interface and the corresponding microstructures at the peritectic interface given in Fig. 2(a1–d1). The dark gray phase, light gray phase and the white phase shown in Fig. 2 correspond to primary Ni_3_Sn_2_ phase, peritectic Ni_3_Sn_4_ phase and the eutectic. What is distinct from our previous researches with constant growth velocity^28^,^29^ is that, obvious detachment of secondary dendrite arms from primary dendrite arms can be observed. Figure 3 shows a typical detachment process of secondary dendrite arms during solidification. The parameters like λ_2_ and root radius of detached arm *R*_*root*_ have been indicated in the experimental pictures of this paper. *S*_*V*_ can not be directly indicated in the experimental pictures, the illustration of *S*_*V*_ can be found in our previous work. The Ni_3_Sn_2_ (α) and Ni_3_Sn_4_ (β) phases have also been indicated in other figures.

It seems that the detachment of secondary arms takes place at temperatures both above and below T_P_ . However, the seemingly “detachment” observed before T_P_ is not the real detachment morphology. The morphologies of the secondary dendrite arms observed before T_P_ are very different from those detached from the primary dendrite stems below T_P_ . The detachment phenomenon can not occur instantaneously; instead, it is a solidification process which takes place gradually. This gradual process can be well reflected from the microstructures below T_P_ . Therefore, since the growth morphology of the real dendrite structure is 3D, the “detachment” observed before T_P_ is most likely to be caused by the section of the sample which shows an irregular view of the sample. This irregular morphology of the dendrites is especially obvious when the growth velocity decreases more quickly since the quick variation in growth velocity makes the structures more inhomogeneous.

**Figure 4:**
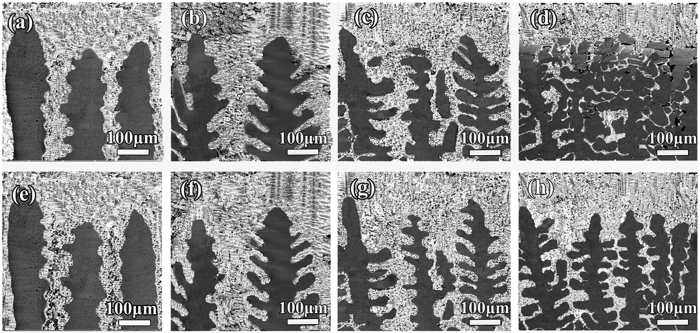
SEM micrographs show the variation of the morphology of the dendritic structures above T_P_ after experiencing successive polishing: (**a–d**) are views of the corresponding structures shown in Fig. 2(a–d) after a polishing of 50 μm; (**e–h**) are views of the corresponding structures shown in Fig. 3(a–d) after a polishing of 50 μm.

But the query still exists in whether the “detachment” phenomenon observed above T_P_ is caused by inhomogeneous dendritic structure. To clarify this, successive sectioning of the dendritc microstructures observed in Fig. 2 which are above T_P_ has been made. As shown in Fig. 4, the total polishing height of the sample increases from (a–d) to (e–h), and the height of every polishing is 50 μm. It can be deduced from the scales in both Figs 2 and 4 that no microstructure feature of detachment of secondary dendrite arms can be observed. Successive sectioning shows that the microstructure does not consist of detached arms, but is one continuous treelike structure of the peritectic Ni_3_Sn_2_ phase, indicating that the influence of peritectic reaction on the detachment process is essentially important in this work. Furthermore, although some alone branches can still be observed after successive sectioning, the development of the detachment process below T_P_ is dominant.

In addition, this detachment can be observed at relatively higher temperatures when the deceleration rate is larger, which means that detachment occurs more quickly and can be more obviously observed at larger deceleration rate. Further observation shows that this detachment is inclined to occur at the thinner secondary dendrite arms which are more likely to dissolve during the detachment process^28^. Both the TGZM and Gibbs-Thomson effects are involved during solidification of this Sn-Ni peritectic alloy^29^. The unsymmetrical distribution of peritectic phase on the secondary dendrite arms is the morphological feature of the TGZM effect in peritectic systems^27^,^29^. It can be seen from Fig. 2(a1–d1) that the peritectic Ni_3_Sn_4_ layer is quite thick at the front edge of the secondary dendrite arm of primary Ni_3_Sn_2_ phase, whereas there is almost no peritectic Ni_3_Sn_4_ phase at the back edge of the secondary dendrite arm of primary Ni_3_Sn_2_ phase. As the temperature decreases further, the Ni_3_Sn_4_ phase at the front edge grows in parallel to the temperature gradient and reaches the back edge of former secondary dendrite arm. In the supplementary material of the present work, the model in which both the Gibbs-Thomson effect and TGZM effect^29^ have been taken into consideration is established for following calculation.

Other interesting features of the microstructure also capture our attentions. The first one is the morphology of the secondary arms itself, which is not uniform or symmetric along the secondary arms, especially for the detached secondary dendrite arms. On the contrary, the tip radii of these secondary arms are obviously larger than the root radii of them in general. The morphologies of these secondary arms are not regular spherical or cylindrical, but are more similar to tear-shaped ones. In addition, this difference between tip and root radius becomes more significant at a lower temperature, indicating that the morphology of the secondary dendrite arms which are inclined to detach is not well established below T_P_ . Another feature can be observed from the comparison with previous work^28^,^29^ at constant growth velocities. Distinct from the microstructures solidified at constant growth velocities, detachment of secondary arms from primary dendrite stems can be more commonly observed in microstructures obtained under deceleration growth condition. These special features indicate that the not only the solute diffusion between thicker and thinner secondary arms, but also that between the tip and root of the thinner arms should be taken into consideration in this work.

### Prediction results

#### Modeling on detachment process in Sn-Ni peritectic system

**Figure 5:**
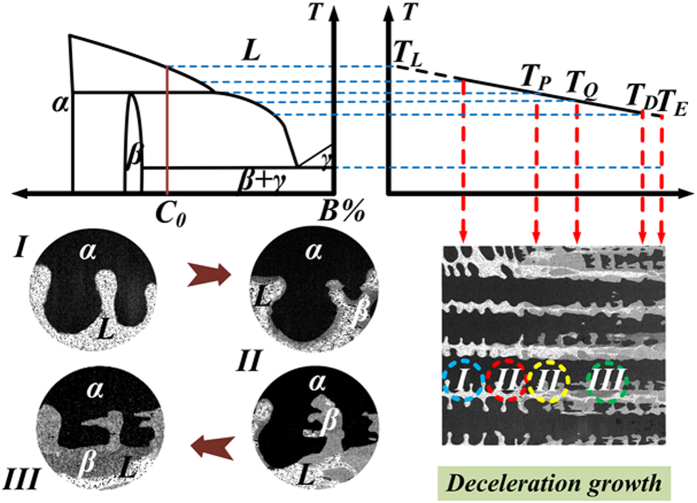
Different stages of detachment of secondary dendrite arm during decelerated directional solidification of Sn-36at.%Ni peritectic alloy: (a) schematic phase diagram of Sn-Ni peritectic alloy; (b) temperature distribution in the direction of temperature gradient: T_L_, T_P_ , T_E_ are the liquidus temperature, peritectic reaction temperature and the eutectic temperature, respectively and T_Q_ is the temperature dividing Stage II and III; (c) schematic drawing of array of both thick and thin dendrite arms; (d) magnified illustrations showing three different stages of the detachment process.

The detachment process shown in Fig. 3 can be divided into different stages in terms of the temperature range during directional solidification of Sn-Ni peritectic alloy. These stages are illustrated in Fig. 5, and the analytical model based on this illustration is proposed to describe this detachment process. The establishment of this analytical model has been presented in detail in the supplementary material. This detachment process is composed of three stages according to both the experimental results and analytical model in the supplementary material. Stage I ranges from T_L_ to T_P_ and stage II ranges from T_P_ to T_Q_, here T_Q_ is the temperature when β phase enclosing the back edge of the root of arm dissolves completely. Stage III is finished when the thinner secondary dendrite dissolves completely. It should be noted that although peritectic transformation also contributes to growth of the peritectic layer, the remelting/resolidification process by TGZM effect and Gibbs-Thomson effect is dominant in determining the thickness of both primary and peritectic phase on the secondary branches. This will be discussed in detail as follows. Besides the ripening process by Gibbs-Thomson effect discussed above, another significant coarsening mechanism is coalescence, which can be seen from the formation of liquid pools included in the coarsened dendrites. However, it can be observed from Figs 2 and 3 that the detachment of the thinner secondary branches has occurred when the volume fraction of liquid is still large in the present work. This indicates that although the coalescence mechanism might be active during the later stage of coarsening process, its influence on the detachment phenomenon is negligible.

The growth/dissolution of peritectic/primary phases occur simultaneously during peritectic transformation. Here query still exists in if the peritectic transformation plays a critical role in this detachment. During peritectic solidification, the peritectic phase usually nucleates on the solid of primary phase, then it grows into both solid and liquid during peritectic interaction. As the diameter of trunk of secondary arm is different, the transformation fraction in radial direction is different. As a matter of fact, since the peritectic phase can precipitate directly from the melt during quenching, the remained secondary arm of primary phase is shown to be surrounded by the peritectic phase. So this growth/dissolution process by peritectic transformation can not lead to detachment of the secondary dendrite arms. It therefore can be concluded that the influence of peritectic transformation on this detachment process can be neglected. On the contrary, the experimental observations (Figs 2 and 3) show the microstructure feature of many other remelting/resolidification mechanisms, like the TGZM effect, the Gibbs-Thomson effect, etc. In addition, the tip radii of these secondary dendrite arms increases during this detachment process while the root radii of them decreases simultaneously, indicating that the curvature difference between the tip and root of secondary dendrite arms also contributes to this detachment process.

In fact, the peritectic transformation occurs immediately after the primary α phase has been enclosed by the peritectic β phase. Therefore, the peritectic transformation also takes place when the remelting/resolidification process by the TGZM effect and Gibbs-Thomson effect proceed. So, it is really very hard to distinguish their influences on the “detachment” of secondary arm. However, it can be found from both experimental prediction and experimental observation that the influence of peritectic transformation on this observed “detachment” of secondary arm is rather limited. The solubility range of peritectic Ni_3_Sn_4_ phase is narrow, so the driving force of growth of peritectic Ni_3_Sn_4_ phase by peritectic transformation is limited. In addition, the diffusion coefficient of atom in solid phases which is only about 1/1,000 of that in liquid phase decreases quickly with decreasing temperatures, which greatly restricts the growth of peritectic Ni_3_Sn_4_ phase through peritectic transformation. From our previous work^33^, the growth thickness of the peritectic β (Ni_3_Sn_4_) phase is this Sn-Ni peritectic alloy is only 5 μm in the vicinity of T_P_ , which is much smaller than the experimental measurement of thickness of peritectic β (Ni_3_Sn_4_) phase. In addition, the solid/liquid interface at the hot side of the liquid pool between secondary dendrite arms is much more zigzag than that at the cold edge of the liquid pool. The zigzag morphology is a feature of TGZM effect, and is produced due to the difference in the remelting velocity. It can be observed from Fig. 2, especially Fig. 2(a1 that the difference of the thickness of different parts of secondary dendrite arm is very inhomogeneous, and this difference is much larger than the growth of peritectic β (Ni_3_Sn_4_) phase by peritectic transformation (5 μm). Based on the above discussion, the “detachment” of secondary arm really occurs during deceleration growth of this Sn-Ni peritectic alloy, and the influence of peritectic transformation on this process can not be overestimated.

### Root radius of secondary dendrite arm during detachment

**Figure 6:**
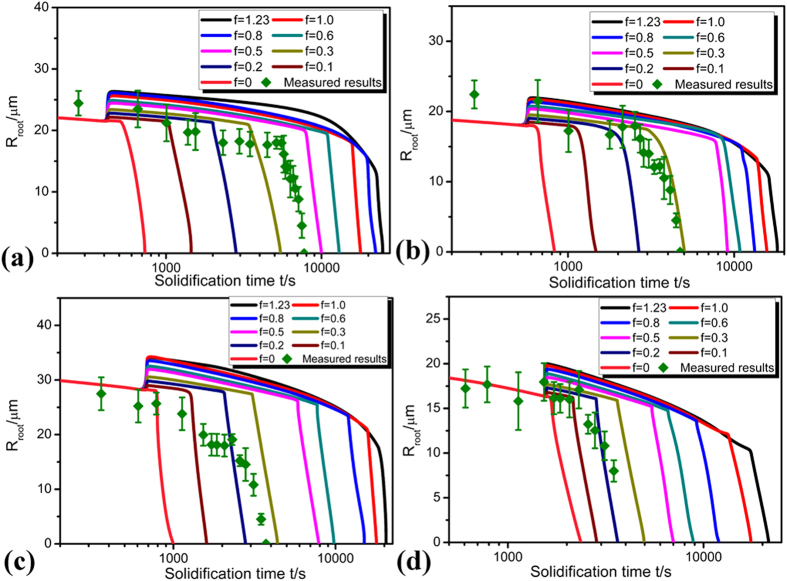
Dependence of the root radius of secondary dendrite arm *R*_*root*_ on solidification time and degree of peritectic reaction at different deceleration rates: **(a)** −2.22 × 10^−10^ m/s^2^, **(b)** −5 × 10^−10^ m/s^2^, **(c)** −8 × 10^−10^ m/s^2^ **(d)** −16 × 10^−10^ m/s^2^.

As shown in Fig. 6, the root radius of detached secondary dendrite arm *R*_*root*_ decreases with increasing solidification time at every deceleration rate. Besides, the dependence of the root radius of secondary dendrite arm during detachment process on degree of peritectic reaction *f* has also been clearly illustrated. The decrease of *R*_*root*_ is restricted by peritectic reaction, which can be observed from the sudden rise of *R*_*root*_; then, as solidification proceeds, *R*_*root*_ continues decreasing. In stage II, decrease of *R*_*root*_ is greatly influenced by degree of peritectic reaction *f*: if peritectic reaction is more complete (*f* is larger), dissolution of peritectic phase at the back edge of the secondary dendrite arms needs more time and stage II is longer. Stage III initiates when the peritectic phase enclosing the back edge of the secondary dendrite arms of primary phase dissolves completely. Both analytical predictions and experimental results indicate that the decrease of *R*_*root*_ is very quick during stage III. The degree of peritectic reaction determined through comparison between analytical predictions and experimental results ranges from 0.1 to 0.4 with decreasing deceleration rates.

### Secondary dendrite arm spacing during detachment

**Figure 7:**
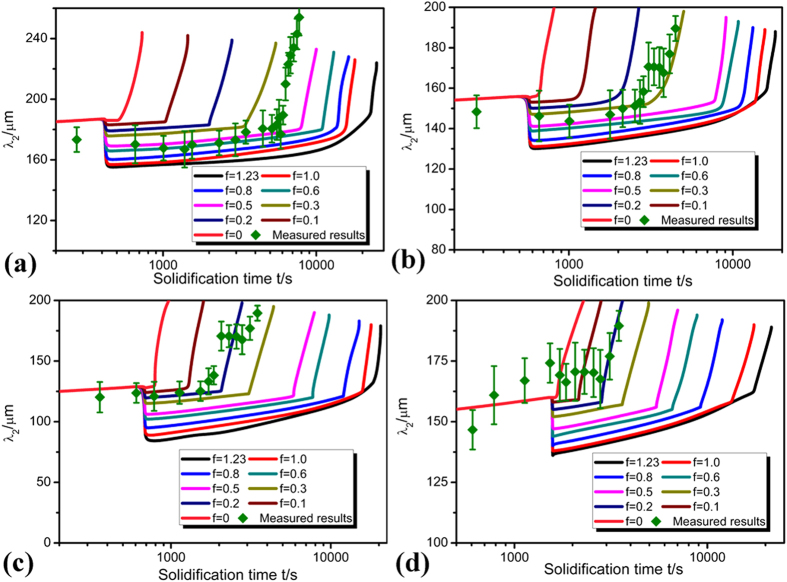
Dependence of the secondary dendrite arm spacing *λ*_*2*_ on solidification time and degree of peritectic reaction at different deceleration rates: **(a)** −2.22 × 10^−10^ m/s^2^, **(b)** −5 × 10^−10^ m/s^2^, **(c)** −8 × 10^−10^ m/s^2^ **(d)** −16 × 10^−10^ m/s^2^.

It can be seen from Fig. 7 that the secondary dendrite arm spacing *λ*_*2*_ increases with increasing solidification time at each deceleration rate. Similar to what has been observed on the root radius *R*_*root*_, the increase of *λ*_*2*_ with increasing solidification time can also be divided into different stages. The sudden decrease of *λ*_*2*_ after peritectic reaction can be clearly observed in both analytical predictions and experimental results, confirming the retard of the detachment process by peritectic reaction. This is consistent with previous researches on coarsening process at constant growth velocities^28^,^29^. Influence of degree of peritectic reaction on *λ*_*2*_ can also be clearly captured from predictions. If peritectic reaction is more complete (*f* is larger), the complete dissolution of the peritectic phase enclosing the back edge of the secondary dendrite arms of primary phase takes a longer period of time. Thus, detachment of the thinner secondary dendrite arms is retarded, and the recurrence of increase of *λ*_*2*_ should be restricted. Quick rise of *λ*_*2*_ can be clearly observed after complete dissolution of the peritectic phase. The degree of peritectic reaction qualified ranges from 0.1 to 0.4 with decreasing deceleration rates, which is very similar to that determined through *R*_*root*_.

### Specific surface area during detachment

**Figure 8:**
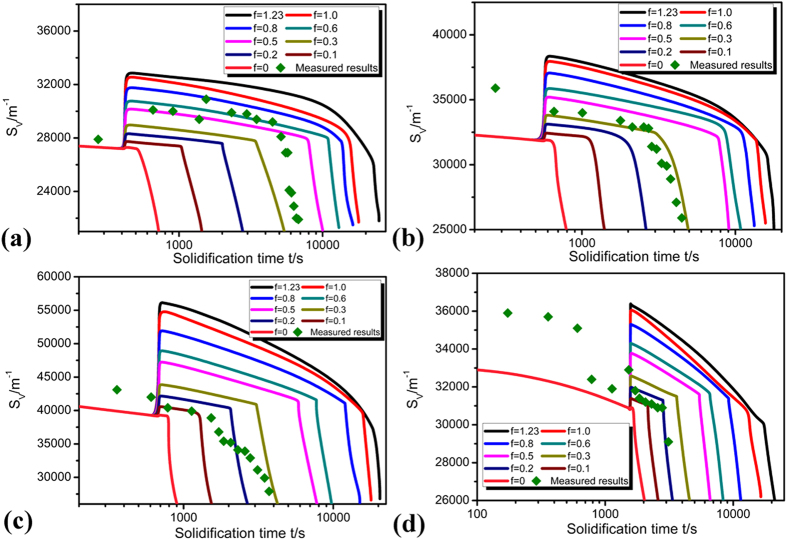
Dependence of the specific surface area *S*_*v*_on solidification time and degree of peritectic reaction at different deceleration rates: **(a)** −2.22 × 10^−10^ m/s^2^, **(b)** −5 × 10^−10^ m/s^2^, **(c)** −8 × 10^−10^ m/s^2^ **(d)** −16 × 10^−10^ m/s^2^.

As shown in Fig. 8, the specific surface area *S*_*V*_ should decrease with increasing solidification time. However, similar to that of *R*_*root*_ and *λ*_*2*_, this decrease is interrupted by peritectic reaction which restricts the detachment process^28^,^29^. Both the initial and terminal values of *S*_*V*_ increases as the deceleration rate increases, which means that the dendritic microstructure is well developed as the growth velocity increases, which brings about larger value of *S*_*V*_. At a given growth velocity, if peritectic constant *f* is small, the peritectic layer enclosing primary Ni_3_Sn_2_ phase is thin and this layer can dissolve quickly, thus, stage II is of short duration. The degree of peritectic reaction determined through *S*_*V*_ matches well with those obtained through *R*_*root*_ and *λ*_*2*_.

## Discussion

It has been demonstrated that remelting at the root of dendrite arms during detachment, and solute accumulation and recalescence could be potent mechanisms for initiation of detachment^34^. However, obvious detachment of secondary dendrites can be observed only below T_P_ , and the duration of thermal recalescence is very short, so there exists no evidence of thermal recalescence in the present experiments. It is therefore concluded that the detachment of secondary dendrite arms must be a solutal effect, either by coarsening or solute accumulation. It is interesting to note that the detachment always takes place on the tear-shaped secondary dendrite arms where the curvatures of tips are obviously larger than those of roots. Dendrites are complex structures with curvature variations along the arms, which determine how the arms will coarsen. The variations in curvature and the corresponding concentration lead to solute fluxes from higher to lower mean curvature regions^1^.

Generally speaking, the local solute concentration varies with the curvature from point to point; and the geometric parameter that influenced the rate of coarsening was the local mean curvature of the interface^1^,^35^,^36^. For this reason, solute fluxes flow between the different parts of the highly ramified dendrites where the local curvature is different. For the tear-shaped dendrite arms, since root radius *R*_*root*_ is much smaller than tip radius *R*_*tip*_, solute diffuses from the tip to the root and gradually accumulates around the roots. The solute supersaturation at the root initiates the remelting of solid phase in an attempt to re-establish the interfacial equilibrium. Some local remelting may be initiated by solvent transport from the root to the tip^8^,^34^,^35^,^37^ even at equilibrium conditions. Then, material is transported from the base to the region near the tip of the arm^34^, thereby decreasing *R*_*root*_ and increasing *R*_*tip*_. This process continues until *R*_*root*_ becomes zero and the tear-shaped arm detaches from the stem of the dendrite.

The discussion above, however, does not consider the tear-shaped interfacial area between the roots of the tear-shaped arms^36^. The tear-shaped interfaces will have one negative radius of curvature, which is small in magnitude. The negatively curved interfaces remain nearly inactive and are almost isolated from other parts of the secondary branches^1^. Thus, although solute transport can be caused by the cylindrical curvature of the narrow root, the kinetics of the detachment process given is unlikely caused by only local curvature difference of secondary branches. The material transport depends not only on local curvature, but also on local solute concentration which fluctuates during solidification. The local curvature is not the unique factor influencing the local solute concentration during solidification. The following content will be focused on the local melt concentration near secondary dendrite arms.

It has been confirmed above that the detachment of secondary dendrite arms was caused by solutal effects during solidification. Due to the curvature difference at different parts of the secondary dendrite arms, solute fluxes between different parts of the secondary branches are produced. Besides the coarsening process by the Gibbs-Thomson effect, other mechanisms such as deceleration of growth velocity^8^,^9^,^20^, the TGZM effect^29^ also contribute to the solutal effect. During deceleration growth, the interdendritic Sn concentration is higher than that under equivalent steady state conditions. It is thus clear that the solute-enriched interdendritic liquid affects the dendritic array by promoting detachment. Due to the negatively curved interfaces remain nearly inactive, the areas around the roots of secondary dendrite arms are relatively isolated as compared with other parts of the secondary branches^1^, and this is convenient for solute accumulation at the roots. The accumulation of solute at the root of secondary branches facilitates remelting by lowering the melting point of the solid–liquid interface. Then detachment takes place from the beginning of necking to the final detachment from the parent dendrite branch.

During cooling in the dendritic array, while the dendrites are coarsening, especially during the earlier stages of ripening, solute (Sn) rejection occurs simultaneously, radially, from primary and secondary arms, with reinforcement of solute at the junctions or nodes. This locally high solute accumulation causes the initial decrease of the root radius of side arms. However, the curvature balance allows these necked roots to persist in equilibrium with the array for a long time, unless either the ambient temperature or the ambient interdendritic solute concentration rises. If the growth velocity *v* and/or temperature gradient *G* changes suddenly, the balance between them becomes so delicate that any slight perturbation can cause almost instantaneous detachment. So, it is the deceleration of dendritic growth front promotes the melting off of side arms is deceleration of the dendritic growth front.

According to the TGZM effect^38^,^39^, a concentration gradient is formed by the temperature gradient, leading to solute fluxes parallel to the temperature gradient. This promotes the accumulation of Sn atoms at the roots of secondary dendrite arms, which accelerates the melting process of the roots, thereby, promoting the detachment of secondary dendrite arms. When extended to peritectic solidification in a temperature gradient, peritectic reaction is closely related to the TGZM effect since two solid phases and the resulting peritectic reaction (Liquid+α→β) is involved. On the one hand, the melting/solidification process induced by the TGZM effect on secondary dendrite arms has been demonstrated to be accelerated by peritectic reaction^29^. On the other hand, the melting/solidification process by the Gibbs-Thomson effect has also been confirmed to be restricted by peritectic reaction^25^,^28^. These effects may have different influences on dendrite coarsening process during peritectic solidification.

The analytical model in the supplementary material shows that the TGZM effect is more important than the Gibbs-Thomson effect in this work. This can also be identified through the experimental observations (Figs 2 and 3). It has been deduced from our measurement that the relations between nucleation undercoolings and cooling velocity *R* of both the primary Ni_3_Sn_2_ phase and peritectic Ni_3_Sn_4_ phase during solidification of Sn-36at.%Ni peritectic alloy are: 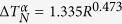 and 
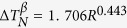
. In consideration of the temperature gradient of 40K/mm of this work, the actual peritectic temperature T_P_ can be deduced from the quenched solid/liquid interface. Thus, the position of T_P_ is determined. The detachment of secondary dendrite arms can be observed below T_P_ only, which means that the remelting by the Gibbs-Thomson effect is not very obvious at the roots of secondary dendrites. Both the detachment and the “sawtooth” like morphology on secondary dendrites result from the melting/solidification process by the TGZM effect. After detachment, the secondary branches still experience the morphological change. The microstructures in Fig. 2 show that the size scales increase dramatically over time and the initially ramified dendritic structure also undergoes morphological changes. Simultaneously, the primary phase becomes distinctly more spheroidal, or globular after long time.

Furthermore, the observations (Figs 2 and 3) show that the morphology of the detached arms is not symmetrical. Besides, the solid/liquid interface at the hot side of the liquid pool between secondary dendrite arms is much more zigzag than that at the cold edge of the liquid pool. According to the TGZM effect, the remelting velocity at the hot side of the liquid pool is larger than the solidification velocity at the cold side of the liquid pool^40^. Thus, the non-symmetrical morphology can be produced. As the morphology of the secondary dendrite arms also influences the remelting/solidification process by the TGZM effect^41^, the remelting/solidification velocities are different at different parts of the secondary dendrites. As a result, the zigzag morphology is produced due to the difference in the remelting velocity^41^. In this case, this zigzag pattern is also accelerated by deceleration growth of the sample. The Sn-enriched interdendritic melt between secondary dendrite arms tend to move to the hot side of the liquid pool during deceleration growth, which might change the local Sn concentration at the solid/liquid interface. Thus, the solute distribution in the liquid pool is more complex and the difference in remelting velocity is more significant, leading to the obviously zigzag morphology.

It is found in our experiment that the reaction constant *f* determined by *S*_*V*_ is in general consistent with those determined by *R*_*root*_ and *λ*_*2*_. The influence of peritectic reaction on the detachment process under deceleration condition can be more clearly understood through analyzing the dependence of *f* on deceleration rate and solidification time. As shown in Figs 6, 7, 8, the values of *f* determined in the present work are not constant but smaller at lower deceleration rates. It can be seen by comparing the predictions and experimental measurements that at lower deceleration rates, the experimental measurements are closer to the prediction when *f* is 0.4, but at higher deceleration rates, they are closer to the prediction when *f* is 0.2. It can therefore be concluded that peritectic reaction is more complete and more peritectic phase are formed at lower deceleration rates. Although it takes longer time for peritectic phase to be dissolved at lower deceleration rates, the proportion of peritectic phase dissolved is smaller in comparison with the peritectic phase formed. Thus, more peritectic phase will be left, and the detachment process will be retarded.

It is worth noting that the range of reaction constant *f* determined by *S*_*V*_ is a little larger than that determined by *λ*_*2*_, which means that the range of peritectic reaction characterized by *S*_*V*_ should be greater than that characterized by *λ*_*2*_ at different growth velocities. On the one hand, the average secondary dendrite arm spacing model uses simplified, approximate interfacial geometry which does not exist for the evolution of branch spacing in dendrites. On the other hand, the driving force for coarsening arises from the free energy associated with the presence of the interface, and *S*_*V*_ has the fundamental physical relevance which can not be so precisely described by *λ*_*2*_ to the coarsening process. So it is very difficult to use *λ*_*2*_ to characterize the coarsening kinetics in the ramified dendritic morphologies, and the characterization by *S*_*V*_ is more comprehensive. However, it should be noted that the difference in reaction constant *f* between that determined by *S*_*V*_ and *λ*_*2*_ under deceleration growth velocity is much smaller than that under constant growth velocity. This is because that this detachment process is directly related to secondary dendrite arm spacing *λ*_*2*_. This means that besides *S*_*V*_, *λ*_*2*_ can also be an appropriate parameter suitable for describing the detachment of secondary branches in the microstructures. Furthermore, it is interesting to note that the value of *f* is not constant even at a given deceleration rate during the real solidification. As directional solidification proceeds, the value of *f* changes, especially at a higher deceleration rate. In fact, dendrite morphology can not be strictly invariant during solidification, and it will be more developed and more easily changed at higher deceleration rates. Consequently, the value of *f* will not be constant at a given deceleration rate in the real solidification process.

The discussions above might be more clearly elucidated using the synchrotron radiation X-ray imaging technique. However, the melting temperature of this Sn-Ni peritectic alloy is much higher than the alloys commonly observed using the synchrotron radiation X-ray imaging technique (Al based alloy etc.). Besides, the dimension of the sample is often special (usually very thin in thickness), which may bring a different solidification environment as compared with this Bridgman-type furnace. So, although there still exists some problems before using the synchrotron radiation X-ray imaging technique, observation of peritectic solidification should be the direction our further effort should be made for. Our further work will be focused on designing experimental procedures to better investigate this phenomenon.

## Conclusion

Directional solidification under deceleration condition have been carried out on Sn-36at.%Ni peritectic alloy in a temperature gradient, and the conclusions are made.

1. Extensive detachment of secondary dendrite arms from primary dendrite stems which could be divided into different stages has been observed at temperatures below T_P_ . The microstructure features show that the Gibbs-Thomson effect, TGZM effect, peritectic reaction, deceleration rate all contribute to this detachment process.
2. An analytical model is established to characterize the detachment process in terms of secondary dendrite arm spacing *λ*_2_, the root radius of detached arms and specific surface area (*S*_*V*_) of dendrites. Analytical predictions match well with experimental results, and the present model has confirmed the contribution of curvature difference between the tip and root of secondary dendrite arms.
3. The detachment of the secondary dendrite arms is obviously accelerated by peritectic reaction through the promotion of the remelting process by the TGZM effect. The kinetics of peritectic reaction which is found to be crucial to determine the detachment process is dependent on both growth velocity and solidification time.

## Experimental Section

Sn-36at.%Ni alloy was induction melted from pure Ni and Sn (99.9%). As-cast rods of 3mm in diameter and 110mm in length were machined by a spark machining from the ingot. Experiments consisting of melting followed by directional solidification were carried out in a Bridgman-type furnace. This Bridgman-type furnace is consisting of a resistance furnace, a water cooled liquid metal bath filled with a liquid Ga-In-Sn alloy, and an adiabatic zone which is located between the heater and the cooler, as previously described^28^,^29^. The samples were 99.99 pct pure alumina crucibles of 4/5.5mm diameter (insider/outside diameter) and length of 150mm. Temperature profiles were measured using a PtRh30-PtRh6 thermocouple inserted down the center of the samples and the temperature gradient close to the solid/liquid interface deduced from the temperature profiles was approximately 40 K/mm. For each experiment, the furnace was heated to 1250 °C to melt the alloy, and then was held for 30 min to homogenize the melt. Subsequently, the samples were solidified at a constant growth velocity for 10 mm and then subjected to controlled deceleration. The deceleration growth continued until a scheduled growth velocity was reached. The solidification of Sn-36at.%Ni peritectic alloy was carried out at different initial growth velocities (3, 5, 5 and 9 μm/s) and the same final growth velocity (1 μm/s). After enough time (9000 s, 8000, 5000 s and 5000 s for the initial growth velocities of 3, 5, 5 and 9 μm/s), the scheduled growth velocity was achieved, then the samples were quenched into liquid Ga-In-Sn alloy quickly to get well the solid/liquid interface.

After the directional solidification, the solidified samples were longitudinally and transversally sectioned, polished and etched with a solution of 10 g FeCl_3_-20ml HCl-180 ml H_2_O for further analysis. In the transverse direction, the sample was serially sectioned along its length using a thin diamond-impregnated blade and then mounted in an epoxy resin such that the cross section (perpendicular to the growth direction) could be prepared for microscopic examination. The microstructures of the samples were revealed and photographed by scanning electron microscopy (SEM (Quanta-200F)). In addition, the energy dispersive spectrometer (EDS) was employed to analyze the solute concentration of phases. Both the secondary dendrite arm spacing λ_2_ and root radius *R*_*root*_ are measured on the longitudinal sections while the specific surface *S*_*V*_is measured on the transverse sections of the sample. The measurements of λ_2_ *S*_*V*_ have been described in detail in our previous work^28^,^29^.

Our measurements are relying on 2-D sections and this may have large error margins if there are only a small number of dendrite arms. However, since extensive detachment of secondary dendrite arms below T_P_ has been observed (as can be seen from the Experimental results Section), there are numerous dendrite arms can be measured. In fact, no less than 15 secondary dendrite arms have been measured at a given temperature. It can therefore be concluded that large error margins can be avoided. The detachment process of secondary dendrite arms from the primary dendrite stem is closely related to secondary dendrite arms themselves. However, this process occurs in the actual 3-D dendritic structures. Thus, different parameters characterizing the detachment process have been used to minimize the error margins. The most appropriate parameter which can describe the variation of 3-D dendritic structure is the specific surface area *S*_*V*_ which can be obtained from transverse sections. Thus, parameters such as *λ*_*2*_ and root radius *R*_*root*_ obtained from longitudinal sections are also appropriate to describe this detachment process.

In order to precisely determine the positions of both T_L_ and T_P_ , the measurement on the nucleation undercooling of both primary Ni_3_Sn_2_ and peritectic Ni_3_Sn_4_ phases were performed by differential scanning clorimetry (DSC-SETARAM) in a continuous mode. The calorimeter was calibrated by measuring the melting temperatures (T_M_) of metallic In, Sn, Al, Au and Pd (99.999 mass % purity). T_M_ was obtained with an accuracy of T_M _± 0.5 °C for all cases. As-cast rods of 8 mm in diameter and length of 10 mm were put into a high-purity alumina crucible of 20 mm length and 10 mm inner diameter. After heating to 1,250 °C at a rate of 10 °C/min, the sample was held for 30 min at this temperature, then the samples were cooled to room temperatureat a range of cooling velocities of 1~100 °C/s. All the experiments were carried out in Ar atmosphere.

## Additional Information

**How to cite this article**: Peng, P. *et al.* Detachment of secondary dendrite arm in a directionally solidified Sn-Ni peritectic alloy under deceleration growth condition. *Sci. Rep.* **6**, 27682; doi: 10.1038/srep27682 (2016).

## Supplementary Material

Supplementary Information
