# Supplementary material for: Detachment of secondary dendrite arm in a directionally solidified Sn-Ni peritectic alloy under deceleration growth condition
Source: Sci Rep. 2016 Jun 8;6:27682. doi: 10.1038/srep27682 (PMC4897705; doi:10.1038/srep27682)
Supplement: Supplementary Information [file srep27682-s1.doc]

**Title: Detachment of secondary dendrite arm in a directionally solidified Sn-Ni peritectic alloy under deceleration growth condition**

**Authors: Peng Penga,b[[1]](#footnote-2)*, Xinzhong Li^c^, JianGong Li^a,b^,Yanqing Su^c^, Jingjie Guo^c^, Hengzhi Fu^c^**

a. Institute of Materials Science and Engineering, Lanzhou University, Lanzhou 730000, China

b. School of Physical Science and Technology, Lanzhou University, Lanzhou 730000, China

c. School of Materials Science and Engineering, Harbin Institute of Technology, Harbin 150001, PR China

**Corresponding author:** Peng Peng

**Affiliation:** Institute of Materials Science and Engineering, Lanzhou University, Lanzhou

150001, PR China

**Postal address:** No. 222, South Tianshui Road, Lanzhou, Gansu zip: 730000

Tel.:+86-931-2166588;

Fax:+86-931-2166588

**E-mail address:** pengp@lzu.edu.cn(P. Peng)

The schematic representation of dendrite morphology to identify different radii at different local positions has been illustrated in **Figure S1**. Referring to this figure, there are as many as five radii to consider, they are: the primary stem *R_1_*, the tip radius of the secondary arm *R_2_*, the root radius of the secondary arm *R_root_*, and that of the concave necks, *R_3_*, and *R_4_*. To keep consistent with the definitions of these radii in previous works^1, 2, 3^, the tip/root radii of the secondary dendrite arm are denoted as *R_2_*/*R_root_*, respectively. Some assumptions are made as follows to establish this analytical model^4^:

(1) Local equilibrium established very rapidly at the solid/liquid interface.

(2) Nucleation undercooling is assumed to be 0.

(3) The radii of the secondary thicker arms are denoted as *R*, and the radii of the tip and root of the thinner arms are denoted as *R_tip_* and *R_root_*, respectively. Both the root and main sections of the dendrite are assumed to be cylindrical.

(4) Due to the Gibbs-Thomson effect, solute diffuses from the thick arm (*R*) to the thin arm along the concentration gradient, the concentration gradient is assumed to be constant and linear.

(5) Due to the Gibbs-Thomson effect between the tip and root of the secondary arm, solute diffuses from the tip (*R_tip_*) to the root (*R_root_*) along the axis of the secondary dendrite arm, and this concentration gradient is also assumed to be constant and linear.

(6) The temperature gradient across the mushy zone is assumed to be constant and opposite to the x-axis, i.e. dT/dx = -G = const, where G >0.

(7) The distribution of melt concentration parallel to the temperature gradient is assumed to follow the liquidus lines. Ni_3_Sn_2_/Ni_3_Sn_4_ liquidus are assumed to be constant straight lines.

(8) The solute fluxes induced by the TGZM and Gibbs-Thomson effect are independent.

(9) The densities of phases involved are assumed to be the same for simplification.

(10) Since the diffusion coefficient in a liquid phase is larger than that in a solid phase by 3–4 orders of magnitude, the solid-state peritectic transformation is neglected.

Different from the coarsening process which has been proved to be more accurately estimated through *S_V_*, the detachment of side arms from primary dendrite stems can be more clearly observed from secondary dendrite arms. Thus, although the advantages of describing the coarsening process by *S_V_* have been clearly listed, the numerical calculation on both the secondary dendrite arm spacing (*λ*_2_)^5^ and specific surface area *S_V_* should be carried out. And the calculation process of both parameters has been given in detail^4, 5^. The degree of completion of peritectic reaction is characterized by a reaction constant *f* ^6^ which is defined as the ratio of the thickness of peritectic layer formed during peritectic reaction to the initial thin-arm radius before peritectic reaction. For Sn–Ni peritectic system, it can be calculated that *f*=0 denotes non-reaction, *f* =1.23 for complete reaction, and 0 < *f* < 1.23 for partial reaction. The physical parameters used in calculations are illustrated in Ref. 7.

**1. Analytical model on root radius of the secondary arm**

Based on the discussion above, when both the Gibbs-Thomson effect and the TGZM effect are taken into consideration, the detachment of secondary branches in a peritectic alloy can be divided into some stages in terms of the temperature ranges during peritectic solidification, as shown in **Figure S2–6**. These stages are: stage I, from T_L_ to T_P_; stage II, from T_P_ to T_Q_; stage III, from T_Q_ to T_D_; stage IV, from T_D_ to T_E_, where T_L_, T_P_ and T_E_ are the liquidus temperature, the peritectic temperature and the eutectic temperature, respectively. T_Q_ is the temperature separating stage II and III, T_D_ is the temperature when the thinner secondary dendrite dissolves completely. T_D_ should be larger than T_E_ since the experimental results show that the detachment has been finished at temperatures much higher than T_E_, thus, only the former three stages really occurs during the detachment process.

**1.1 Stage I**

As shown in **Figure S2**, in the initial stage ranging from T_L_ to T_P_ [A–A view in Figure 4(d) in the manuscript], only primary α phase is involved. The relevant part of the phase diagram of a binary peritectic system above T_P_ is illustrated in Figure S2. The distribution of solute concentration in the interdendritic liquid phase is also presented. The temperatures of the edges of dendrite arms are denoted as T_1_ and T_2_, respectively; the oblique straight lines shown in Figure S2 are the liquidus lines of primary phase. Considering the reduction of liquidus temperature due to the Gibbs-Thomson effect, the liquidus moves up when the radius of the secondary dendrite arm is *R_root_*. The circles in Figure S2 stand for the morphology of these three secondary dendrite arms along the direction of secondary dendrite arms.

The undercooling due to curvature difference between the tip and root of the secondary dendrite arm is^3^:

$\Delta T_{R}=\Gamma\left(\frac{1}{R_{root}}-\frac{1}{R_{3}}\right)-\frac{1}{R_{2}}$ (1a)

Since the distance between the tip and root of the secondary dendrite arm along the axis of secondary branches is much larger than that along the direction of temperature gradient, thus, the diffusion flux between the tip and root is assumed to be only caused by curvature difference. And the diffusion flux between the tip and root due to temperature gradient is neglected. Considering the initial coarsening by referring to Figure S2, one has:

$T_{1}^{'}=T_{\alpha}+m_{l}^{\alpha}C_{l}^{\alpha,T_{1}^{'}}-\frac{\Gamma}{R}$ (S1b)

$T_{1}=T_{\alpha}+m_{l}^{\alpha}C_{l}^{\alpha,T_{1}}-\Gamma\left[\left(\frac{1}{R_{root}}-\frac{1}{R_{3}}\right)-\frac{1}{R_{2}}\right]$ (S1c)

where *Γ* is the Gibbs-Thomson coefficient; *D* is the diffusion coefficient of solute in liquid; $m_{l}^{\alpha}$is the liquidus slope of primary α phase; $C_{l}^{\alpha,T_{1}^{'}}$and $C_{l}^{\alpha,T_{1}}$are the melt concentrations at T_1_′ and T_1_, respectively. Simultaneously, due to assumptions 2 and 3, concentration gradient resulting from temperature gradient is established within the interdendritic liquid layer:

$T_{1}^{'}=T_{1}+GL_{a}$ (S1d)

Combining the above three equations:

$C_{l}^{\alpha,T_{1}}-C_{l}^{\alpha,T_{1}^{'}}=\frac{\Gamma}{m_{l}^{\alpha}}\left[\left(\frac{1}{R_{root}}-\frac{1}{R_{3}}\right)-\frac{1}{R_{2}}-\frac{1}{R}\right]-\frac{GL_{a}}{m_{l}^{\alpha}}$ (S2)

The section $\frac{\Gamma}{m_{l}^{\alpha}}\left[\left(\frac{1}{R_{root}}-\frac{1}{R_{3}}\right)-\frac{1}{R_{2}}-\frac{1}{R}\right]$ corresponds to the influence of the Gibbs-Thomson effect on the root radius of secondary dendrite arm. The section $\frac{GL_{a}}{m_{l}^{\alpha}}$ corresponds to the TGZM effect. And it can be obtained from Equation S2 that when the TGZM effect is important in comparison with the Gibbs-Thomson effect, there exists $GL_{a}>\Gamma\left[\left(\frac{1}{R_{root}}-\frac{1}{R_{3}}\right)-\frac{1}{R_{2}}-\frac{1}{R}\right]$. In the present work, *R*, *R_2_*, *R_3_* and *R_root_* are of the order of 10^-5^m and the Gibbs-Thomson coefficient is of the order of 10^-7^Km. Thus, the TGZM effect which is more important is of the order of 10^4^K/m. In this case, the melting/solidification process driven by the TGZM effect greatly restricts that by the Gibbs-Thomson effect.

It can also be seen from Figure S2 that the solute concentration difference caused by the Gibbs-Thomson effect across these two liquid layers are$\left|C_{l}^{\alpha,T_{1}}-C_{l}^{r,T_{1}}\right|$ and$\left|C_{l}^{\alpha,T_{2}}-C_{l}^{r,T_{2}}\right|$ while that caused by the TGZM effect are$\left|C_{l}^{r,T_{1}}-C_{l}^{r,T_{1}^{'}}\right|$ and $\left|C_{l}^{r,T_{2}^{'}}-C_{l}^{r,T_{2}}\right|$. Therefore, the solute concentration difference across these two liquid layers are $\left|C_{l}^{r,T_{1}}-C_{l}^{\alpha,T_{1}^{'}}\right|$and$\left|C_{l}^{\alpha,T_{2}^{'}}-C_{l}^{r,T_{2}}\right|$ in non-isothermal condition. And it can be concluded that the TGZM effect is important as compared with the Gibbs-Thomson effect, which is consistent with the discussion above. This indicates that the coarsening process by the Gibbs-Thomson effect is restricted/accelerated by the TGZM effect at the front/back edges of the thinner secondary dendrite arm, respectively. Therefore, due to the coupling effects of the Gibbs-Thomson and TGZM effects, solidification/dissolution occurs at the front/back edges of the thinner secondary dendrite arm.

According to the Fick’s First law:

$J_{1}=D\frac{C_{l}^{\alpha,T_{1}}-C_{l}^{\alpha,T_{1}^{'}}}{L_{a}}=\frac{D}{m_{l}^{\alpha}L_{a}}\left[\Gamma\left[\left(\frac{1}{R_{root}}-\frac{1}{R_{3}}\right)-\frac{1}{R_{2}}-\frac{1}{R}\right]-GL_{a}\right]$ (S3)

The application of a mass balance for a small displacement *dR_root_* of the α/liquid interface results in the following differential equation:

$J_{2}=C_{l}^{\alpha,T_{1}}\left(1-k_{\alpha}\right)\left.\frac{dR_{root}}{dt}\right|_{T_{1}}$ (S4)

$J_{1}=J_{2}$ (S5)

$\left.\frac{dR_{root}}{dt}\right|_{T_{1}}=\frac{D}{m_{l}^{\alpha}C_{l}^{\alpha,T_{1}}\left(1-k_{\alpha}\right)L_{a}}\left[\Gamma\left[\left(\frac{1}{R_{root}}-\frac{1}{R_{3}}\right)-\frac{1}{R_{2}}-\frac{1}{R}\right]-GL_{a}\right]$ (S6a)

As has been discussed above, the temperature gradient is high enough that it restricts the dissolution by the Gibbs-Thomson effect which occurs at the front edge of the root. Thus, the solidification of primary α phase induced by the TGZM effect occurs on the front edge of the root of secondary dendrite arm. Similarly, for the back edge of the root of secondary dendrite arm:

$\left.\frac{dR_{root}}{dt}\right|_{T_{2}}=\frac{D}{m_{l}^{\alpha}C_{l}^{\alpha,T_{2}}\left(1-k_{\alpha}\right)L_{b}}\left[\Gamma\left[\left(\frac{1}{R_{root}}-\frac{1}{R_{3}}\right)-\frac{1}{R_{2}}-\frac{1}{R}\right]+GL_{b}\right]$ (S6b)

where $C_{l}^{\alpha,T_{2}}$and $C_{l}^{\alpha,T_{2}^{'}}$are the melt concentrations at the α/liquid interface with temperatures T_2_ and T_2_′, respectively. In this condition, the TGZM effect accelerates the dissolution by the Gibbs-Thomson effect which occurs at the back edge of the secondary dendrite arms. By referring to assumptions 2–3, the relation between $C_{l}^{\alpha,T_{1}}$and $C_{l}^{\alpha,T_{2}}$is:

$C_{l}^{\alpha,T_{1}}-C_{l}^{\alpha,T_{2}}=\frac{G}{m_{l}^{\alpha}}\cdot\left(2R_{root}\right)$ (S7)

$\left.\frac{dR_{root}}{dt}\right|_{stageI}=\frac{1}{2}\left(\left.\frac{dR_{root}}{dt}\right|_{T_{1}}+\left.\frac{dR_{root}}{dt}\right|_{T_{2}}\right)$ (S8)

Comparison between Equation (6a) and (6b) shows that the dissolution rate at T_2_ is larger than the solidification rate at T_1_, thus, the root radius of secondary dendrite arm gradually decreases as directional solidification proceeds. In conclusion, the Gibbs-Thomson effect and the TGZM effect have opposite/identical influence on the detachment process at different edges of the thinner dendrite arm.

**1.2 Stage II**

When peritectic reaction occurs below T_P_, β phase forms and quickly envelopes the secondary dendrite arm of primary α phase. As shown in **Figure S3**, β phase which forms at the front/back edges of the root of secondary dendrite arm is located at T_1_/T_2_ (T_1_>T_2_). In this case, as shown in Figure S3, within the liquid layer ranging from T_1_′ to T_2_′, the solute concentration difference by the TGZM effect is larger than that by the Gibbs-Thomson effect. Thus, similar to what has been discussed in stage I, the TGZM effect restricts the dissolution by the Gibbs-Thomson effect which occurs at the front edge of the root of secondary dendrite arm. Therefore, the solidification of peritectic β phase induced by the TGZM effect occurs at the front edge of the root of dendrite arm. Besides, the TGZM effect accelerates the dissolution by the Gibbs-Thomson effect which occurs at the back edge of the root. As a result, β phase should solidify/dissolve at T_1_/T_2_. The relevant part of the phase diagram of a binary peritectic system below T_P_ is illustrated in Figure S3, the corresponding solute concentration distribution in phases has also been presented. Similar to what has been proposed in stage I, at T_1_:

$\left.\frac{dR_{root}}{dt}\right|_{T_{1}}=\frac{D}{m_{l}^{\beta}C_{l}^{\beta,T_{1}}\left(1-k_{\beta}\right)L_{a}}\left[\Gamma\left[\left(\frac{1}{R_{root}}-\frac{1}{R_{3}}\right)-\frac{1}{R_{2}}-\frac{1}{R}\right]-GL_{a}\right]$ (S9a)

In stage II, the dissolution rate of β phase at T_2_ can be given by:

$\left.\frac{dR_{root}}{dt}\right|_{T_{2}}=\frac{D}{m_{l}^{\beta}C_{l}^{\beta,T_{2}}\left(1-k_{\beta}\right)L_{b}}\left[\Gamma\left[\left(\frac{1}{R_{root}}-\frac{1}{R_{3}}\right)-\frac{1}{R_{2}}-\frac{1}{R}\right]+GL_{b}\right]$ (S9b)

where $m_{l}^{\beta}$is the liquidus slope of peritectic β phase, $C_{l}^{\beta,T_{1}}$and $C_{l}^{\beta,T_{2}}$are the solute concentrations of liquid at the β/liquid interface at T_1_ and T_2_, respectively. The relation between $C_{l}^{\beta,T_{1}}$and $C_{l}^{\beta,T_{2}}$is:

$C_{l}^{\beta,T_{1}}-C_{l}^{\beta,T_{2}}=\frac{G}{m_{l}^{\beta}}\cdot\left(2R_{root}\right)$ (S10)

$\left.\frac{dR_{root}}{dt}\right|_{stageII}=\frac{1}{2}\left(\left.\frac{dR_{root}}{dt}\right|_{T_{1}}+\left.\frac{dR_{root}}{dt}\right|_{T_{2}}\right)$ (S11)

Furthermore, it should be noted that the rate of the peritectic reaction is important because the "β-layer" thickness is determined, on the one hand, by the rate of the peritectic reaction, and, on the other hand, by the rate of dissolving this "β-layer" due to the coupling influence of the TGZM and G-T effects. Besides, the peritectic reaction rate depends on both the "β-layer" temperature and the "β-layer" thickness which are time-dependent. Therefore, it is necessary to compare the peritectic reaction rate and the dissolution rate of β phase by the coupling influence of the TGZM and G-T effects.

As the nucleation undercooling is negligible, thus, at the initial of stage II, when peritectic reaction occurs, the β phase encloses the root of primary phase instantaneously ^[7]^. If the temperature of the β phase at back edge of the root of secondary dendrite arm is T_2_, and the temperature of α phase at the back edge of the root of secondary dendrite arm is denoted as T_α2_, then it can be obtained that:

$T_{\alpha2}=T_{2}+G\Delta$ (S12)

where *∆*is the thickness of the β phase enclosing the thinner secondary dendrite arm.

It has been proposed that the driving force for the peritectic reaction between T_2_ and T_α2_ is the melt concentration difference $C_{l}^{\beta,T_{2}}-C_{l}^{\alpha,T_{\alpha2}}$^8^. Thus, the solute flux between T_2_ and T_α2_ is:

$J_{P}=D\frac{C_{l}^{\beta,T_{2}}-C_{l}^{\alpha,T_{\alpha2}}}{\Delta}$ (S13a)

And the solute flux at T_2_ due to the TGZM+GT effects is:

$J_{TG}=D\frac{C_{l}^{\beta,T_{2}^{'}}-C_{l}^{\beta,T_{2}}}{L_{b}}$ (S13b)

Thus, the comparison between the peritectic reaction rate and the β phase dissolution rate by the TGZM+GT effects has changed into comparing the solute fluxes presented above. It can be obtained that:

$\begin{matrix} C_{l}^{\beta,T_{2}^{'}}-C_{l}^{\beta,T_{2}}=\frac{T_{P}-T_{2}}{m_{l}^{\beta}}-\frac{T_{P}-T_{2}^{'}}{m_{l}^{\beta}} \\ =\frac{T_{2}^{'}-T_{2}}{m_{l}^{\beta}}=\frac{GL_{b}}{m_{l}^{\beta}} \end{matrix}$ (S14a)

$\begin{matrix} C_{l}^{\beta,T_{2}}-C_{l}^{\alpha,T_{\alpha2}}=\frac{T_{P}-T_{\alpha2}}{m_{l}^{\alpha}}-\frac{T_{P}-T_{2}}{m_{l}^{\beta}} \\ =\left(T_{P}-T_{2}\right)\left(\frac{1}{m_{l}^{\alpha}}-\frac{1}{m_{l}^{\beta}}\right)-\frac{Gl}{m_{l}^{\alpha}} \end{matrix}$ (S14b)

Therefore, it can be obtained through Equation S13 and S14 that:

$\left|\frac{J_{P}}{J_{TG}}\right|=\left|\frac{GL}{m_{l}^{\beta}}\right|/\left|\left(T_{P}-T_{2}\right)\left(\frac{1}{m_{l}^{\alpha}}-\frac{1}{m_{l}^{\beta}}\right)-\frac{Gl}{m_{l}^{\alpha}}\right|$ (S15)

Here $\left|L_{b}\right|$ is much larger than $\left|\Delta\right|$, and based on the experimental results, it is assumed that$\left|L\right|\approx10\left|\Delta\right|$. Futhermore, $\left|m_{l}^{\beta}\right|\approx2.2\left|m_{l}^{\alpha}\right|$, thus it can be obtained that there always exists . Besides, as shown in Equation S15, the value of$\left(T_{P}-T_{2}\right)$ increases during the process of directional solidification, the value of $\left|\frac{J_{P}}{J_{TG}}\right|$ continues increasing. Thus, it can be concluded that the solidification rate by peritectic reaction is smaller than the dissolution rate of β phase by the coupling influence of the TGZM and G-T effects, and this difference increases as solidification proceeds. In this case, the rate of peritectic reaction is not taken into account in stage II. As the dissolution rate at T_2_ is larger than the solidification rate at T_1_, β phase which previously forms at T_2_ dissolves completely first at temperature T_Q_, then stage III initiates.

**1.3 Stage III**

If β phase enclosing the back edge of the root of arm dissolves completely first, both α and β phases are involved in the detachment process. As shown in **Figure S4**, the oblique solid straight lines above/below T_P_ are the liquidus lines of primary/peritectic phase. The oblique dashed straight lines below T_P_ are the extended liquidus lines of primary phase. In this case, as shown in Figure S4a, within the liquid layer ranging from T_1_′ to T_1_, the TGZM effect is important according to the discussion in Section 1.1. For the liquid layer with temperatures ranging from T_2_ to T_2_′, as shown in Figure S4a, the TGZM effect is also important. Thus β phase should solidify at T_1_ and α phase should dissolve at T_2_.

Thus, for the front edge, similar to what has been proposed in stage II, one has:

$\left.\frac{dR_{root}}{dt}\right|_{T_{1}}=\frac{D}{m_{l}^{\beta}C_{l}^{\beta,T_{1}}\left(1-k_{\beta}\right)L_{a}}\left[\Gamma\left[\left(\frac{1}{R_{root}}-\frac{1}{R_{3}}\right)-\frac{1}{R_{2}}-\frac{1}{R}\right]-GL_{a}\right]$ (S16)

where $C_{l}^{\beta,T_{1}}$is the solute concentration of liquid at the β/liquid interface at T_1_. For the back edge of root of dendrite arm, one has:

$T_{2}^{'}=T_{\beta}+m_{l}^{\beta}C_{l}^{\beta,T_{2}^{'}}-\frac{1}{R}$ (S17a)

$T_{2}=T_{\alpha}+m_{l}^{\alpha}C_{l}^{\alpha,T_{2}}-\Gamma\left[\left(\frac{1}{R_{root}}-\frac{1}{R_{3}}\right)-\frac{1}{R_{2}}\right]$ (S17b)

Simultaneously, due to assumptions 2 and 3, concentration gradient resulting from the temperature gradient is established within the interdendritic liquid layer:

$T_{2}=T_{2}^{'}+GL_{b}$ (S17c)

Combining the above three equations:

$C_{l}^{\beta,T_{2}^{'}}-C_{l}^{\alpha,T_{2}}=\frac{1}{m_{l}^{\beta}}\left[\left(m_{l}^{\alpha}-m_{l}^{\beta}\right)\left(C_{l}^{\alpha,T_{2}}-C_{LP}\right)-\Gamma\left[\left(\frac{1}{R_{root}}-\frac{1}{R_{3}}\right)-\frac{1}{R_{2}}-\frac{1}{R}\right]-GL_{b}\right]$ (S18)

A flux is induced by the composition gradient across the liquid with temperatures ranging from T_2_ to T_2_′:

$J=D\frac{C_{l}^{\beta,T_{2}^{'}}-C_{l}^{\alpha,T_{2}}}{L_{b}}$ (S19)

By applying a mass balance for a small displacement *dR_root_* of the α/liquid interface,

$J=D\frac{C_{l}^{\beta,T_{2}^{'}}-C_{l}^{\alpha,T_{2}}}{L_{b}}=\frac{dR_{root}}{dt}\left(C_{\alpha}^{l,T_{2}}-C_{l}^{\alpha,T_{2}}\right)$ (S20)

Combining the above equations, the dissolution rate of α phase at T_2_ is:

$\left.\frac{dR_{root}}{dt}\right|_{T_{2}}=\frac{D}{m_{l}^{\beta}C_{l}^{\alpha,T_{2}}L_{b}\left(1-k_{\alpha}\right)}\left[\Gamma\left[\left(\frac{1}{R_{root}}-\frac{1}{R_{3}}\right)-\frac{1}{R_{2}}-\frac{1}{R}\right]+GL_{b}+\left(m_{l}^{\alpha}-m_{l}^{\beta}\right)\left(C_{LP}-C_{l}^{\alpha,T_{2}}\right)\right]$ (S21)

where $C_{l}^{\alpha,T_{2}}$is the solute concentration of liquid at the α/liquid interface at T_2_. The relation between $C_{l}^{\beta,T_{1}}$in Equation S10 and$C_{l}^{\alpha,T_{2}}$is:

$\begin{matrix} C_{l}^{\alpha,T_{2}}-C_{l}^{\beta,T_{1}}=\frac{T_{P}-T_{1}}{m_{l}^{\beta}}-\frac{T_{P}-T_{2}}{m_{l}^{\alpha}} \\ =\left(T_{P}-T_{1}\right)\left(\frac{1}{m_{l}^{\beta}}-\frac{1}{m_{l}^{\alpha}}\right)-\frac{G}{m_{l}^{\alpha}}\cdot\left(2R_{root}\right) \end{matrix}$ (S22)

$\left.\frac{dR_{root}}{dt}\right|_{stageIII}=\frac{1}{2}\left(\left.\frac{dR_{root}}{dt}\right|_{T_{1}}+\left.\frac{dR_{root}}{dt}\right|_{T_{2}}\right)$ (S23)

Similar to what has been proposed in stage II, it can also be obtained that the peritectic reaction rate is smaller than the β phase dissolution rate by the TGZM+GT effects, and this difference increases as solidification proceeds. As a result, the rate of the peritectic reaction is not taken into account in stage III. It can be found from Equation S21 that the dissolution rate increases with the increase of$C_{l}^{\alpha,T_{2}}$. According to Equation S22, $C_{l}^{\alpha,T_{2}}$increases with increasing temperature gradient *G* but decreases with the decrease of temperature at the β/liquid interface T_1_. This indicates that the dissolution rate of α phase can be significantly accelerated if the temperature gradient increases.

As the detachment process proceeds, as shown in Figure S4b, β/α phase at T_1_′/T_2_ gradually dissolve. If peritectic reaction is not very complete, then β phase enclosing the secondary arm of primary α phase is not thick. Thus, as shown in Figure S4b, β phase at T_1_′ dissolves completely before complete dissolution of α phase at T_2_. Otherwise, if peritectic reaction is more complete, then β phase enclosing the thicker secondary arm of primary α phase is thicker. In this case, α phase at T_2_ dissolves completely before complete dissolution of β phase at T_1_′. Thus it can be concluded that the degree of completion of peritectic reaction is of noticeable importance for the detachment process. However, in both cases, β phase at T_1_′should dissolve completely as the detachment process proceeds. No matter how complete peritectic reaction is, the root of secondary dendrite arm is composed of peritectic β phase while the edges facing it are of α/β phase at T_1_′/T_2_′, as illustrated in Figure S4c. In this case, similar to what has been proposed in Equation S21, the solidification rate of the peritectic β phase at T_1_ in stage IV is:

$\left.\frac{dR_{root}}{dt}\right|_{T_{1}}=\frac{D}{m_{l}^{\alpha}C_{l}^{\beta,T_{1}}\left(1-k_{\beta}\right)L_{a}}\left[\left(m_{l}^{\beta}-m_{l}^{\alpha}\right)\left(C_{LP}-C_{l}^{\beta,T_{1}}\right)+\Gamma\left[\left(\frac{1}{R_{root}}-\frac{1}{R_{3}}\right)-\frac{1}{R_{2}}-\frac{1}{R}\right]-GL_{a}\right]$(S24a)

where $C_{l}^{\beta,T_{1}}$is the solute concentration of liquid at the β/liquid interface with the temperature T_1_. For the back edge of root of dendrite arm, the dissolution rate of β phase at T_2_ is:

$\left.\frac{dR_{root}}{dt}\right|_{T_{2}}=\frac{D}{m_{l}^{\beta}C_{l}^{\beta,T_{2}}\left(1-k_{\beta}\right)L_{b}}\left[\Gamma\left[\left(\frac{1}{R_{root}}-\frac{1}{R_{3}}\right)-\frac{1}{R_{2}}-\frac{1}{R}\right]+GL_{b}\right]$ (S24b)

Here the relation between $C_{l}^{\beta,T_{1}}$and$C_{l}^{\beta,T_{2}}$ is:

$C_{l}^{\beta,T_{1}}-C_{l}^{\beta,T_{2}}=\frac{G}{m_{l}^{\beta}}\cdot\left(2R_{root}\right)$ (S25)

$\left.\frac{dR_{root}}{dt}\right|_{stageIV}=\left.\frac{dR_{root}}{dt}\right|_{T_{1}}+\left.\frac{dR_{root}}{dt}\right|_{T_{2}}$ (S26)

When peritectic β phase dissolves completely at both T_1_ and T_2_, as shown in Figure S4d, the front edges of two thicker secondary dendrite arms are both enclosed by peritectic β phase. Comparison between the schematic illustration in Figure S4d and the experimental results in Figure S2 of the manuscript shows that the ultimate state in Figure S4d in the manuscript is nearly the same as the experimental results. Thus, the contribution to detachment process by the Gibbs-Thomson effect is smaller than that by the TGZM effect when both the primary and peritectic phases are involved. Once the liquid phase between the two secondary dendrite arms is consumed completely, further peritectic transformation can take place by solid-state diffusion through β phase, which will not be discussed in this work.

**2. Analytical model on tip radius of the secondary arm**

As illustrated in Equation S6, S9, S16, S21 and S24, dependence of the root radius of the secondary dendrite arm *R_root_* on the tip radius of the tear-shaped secondary dendrite arm *R_2_* shows that the variation of *R_2_* during the detachment process should also be identified. As can be observed from Figure S2 and S3 in the manuscript, the tip radii of the secondary dendrite arms detached from primary stem are in general smaller than those of their neighboring thicker ones, namely, *R_2_*˂*R*. Similarly to what has been discussed on the root radius of detached secondary dendrite arm, the variation of the tip radius of secondary dendrites can also be described in terms of temperature ranges during peritectic solidification, as shown in Figure S5–S7.

**2.1 Stage I**

As shown in **Figure S5**, in the initial stage ranging from T_L_ to T_P_, only primary α phase is involved. The relevant part of the phase diagram of a binary peritectic system above T_P_ is illustrated in Figure S5, the distribution of solute concentration in the interdendritic liquid phase is also presented. The temperatures of the edges of dendrite arms are denoted as T_R1_ and T_R2_, respectively; the oblique straight lines shown in Figure S5 are the liquidus lines of primary phase. The circles in Figure S5 stand for the morphology of these three secondary dendrite arms along the direction of secondary dendrite arms. The arrows in Figure S5 indicate the flux directions: from greater concentrations to smaller ones.

The undercooling due to curvature effect is^3^:

$\Delta T_{R}=\Gamma\left(\frac{1}{R_{root}}-\frac{1}{R_{3}}\right)-\frac{1}{R_{2}}$ (S27a) Thus, while the actual temperature at the root of the detached secondary dendrite arm is lower than that under equilibrium condition, the actual temperature at the tip of the detached secondary dendrite arm is higher than it. Considering the initial coarsening by referring to Figure S5, one has:

$T_{1}^{'}=T_{\alpha}+m_{l}^{\alpha}C_{l}^{\alpha,T_{1}^{'}}-\frac{\Gamma}{R}$ (S27b)

$T_{R1}=T_{\alpha}+m_{l}^{\alpha}C_{l}^{\alpha,T_{R1}}+\Gamma\left[\left(\frac{1}{R_{root}}-\frac{1}{R_{3}}\right)-\frac{1}{R_{2}}\right]$ (S27c)

where *Γ* is the Gibbs-Thomson coefficient; *D* is the diffusion coefficient of solute in liquid; $m_{l}^{\alpha}$is the liquidus slope of primary α phase; $C_{l}^{\alpha,T_{1}^{'}}$and $C_{l}^{\alpha,T_{R1}}$are the melt concentrations at T_1_′ and T_R1_, respectively. Simultaneously, due to assumptions 2 and 3, concentration gradient resulting from temperature gradient is established within the interdendritic liquid layer:

$T_{1}^{'}=T_{R1}+GL_{Ra}$ (S27d)

Combining the above three equations:

$C_{l}^{\alpha,T_{R1}}-C_{l}^{\alpha,T_{1}^{'}}=-\frac{\Gamma}{m_{l}^{\alpha}}\left[\left(\frac{1}{R_{root}}-\frac{1}{R_{3}}\right)-\frac{1}{R_{2}}-\frac{1}{R}\right]-\frac{GL_{Ra}}{m_{l}^{\alpha}}$ (S28)

Based on our discussion above, the melting/solidification process driven by the TGZM effect greatly restricts that by the Gibbs-Thomson effect.

It can also be seen from Figure S5 that the solute concentration difference caused by the Gibbs-Thomson effect across these two liquid layers are $\left|C_{l}^{R,T_{R1}}-C_{l}^{\alpha,T_{R1}}\right|$ and $\left|C_{l}^{R,T_{R2}}-C_{l}^{\alpha,T_{R2}}\right|$ while that caused by the TGZM effect are $\left|C_{l}^{\alpha,T_{R1}}-C_{l}^{\alpha,T_{1}^{'}}\right|$ and $\left|C_{l}^{\alpha,T_{2}^{'}}-C_{l}^{\alpha,T_{R2}}\right|$. Therefore, the solute concentration difference across these two liquid layers are $\left|C_{l}^{R,T_{R1}}-C_{l}^{\alpha,T_{1}^{'}}\right|$and $\left|C_{l}^{\alpha,T_{2}^{'}}-C_{l}^{R,T_{R2}}\right|$in non-isothermal condition. And it can be concluded that the TGZM effect is important as compared with the Gibbs-Thomson effect, which is consistent with the discussion above. This indicates that the coarsening process by the Gibbs-Thomson effect is restricted/accelerated by the TGZM effect on the front/back edges of the thinner secondary dendrite arm, respectively. Therefore, due to the coupling influence of the Gibbs-Thomson effect and the TGZM effect, solidification/dissolution occurs on the front/back edges of the thinner secondary dendrite arm. Furthermore, it should be noted that the solute concentration difference caused by the Gibbs-Thomson effect is smaller as compared with that on the root of the secondary dendrite arm. This is consistent with that the difference in radius between the tip of the thinner secondary branch and its neighboring thicker ones is smaller than that at the root of the thinner secondary branch. For this reason, solidification which occurs at the front edge of the secondary dendrite arm is weakened while remelting which occurs at the back edge of the secondary dendrite arm is enhanced.

According to the Fick’s First law:

$J_{1}=D\frac{C_{l}^{\alpha,T_{R1}}-C_{l}^{\alpha,T_{1}^{'}}}{L_{Ra}}=-\frac{D}{m_{l}^{\alpha}L_{Ra}}\left[\Gamma\left[\left(\frac{1}{R_{root}}-\frac{1}{R_{3}}\right)-\frac{1}{R_{2}}-\frac{1}{R}\right]+GL_{Ra}\right]$ (S29)

The application of a mass balance for a small displacement *dR_2_* of the α/liquid interface results in the following differential equation:

$J_{2}=C_{l}^{\alpha,T_{R1}}\left(1-k_{\alpha}\right)\left.\frac{dR_{2}}{dt}\right|_{T_{R1}}$ (S30)

$J_{1}=J_{2}$ (S31)

$\left.\frac{dR_{2}}{dt}\right|_{T_{R1}}=-\frac{D}{m_{l}^{\alpha}C_{l}^{\alpha,T_{R1}}\left(1-k_{\alpha}\right)L_{Ra}}\left[\Gamma\left[\left(\frac{1}{R_{root}}-\frac{1}{R_{3}}\right)-\frac{1}{R_{2}}-\frac{1}{R}\right]+GL_{Ra}\right]$ (S32a)

As has been discussed above, the temperature gradient value is high enough in this condition. As a result, the TGZM effect accelerates/restricts the solidification of the tip at the front/back edge induced by the Gibbs-Thomson effect. Similarly, for the back edge of the tip of dendrite arm:

$\left.\frac{dR_{2}}{dt}\right|_{T_{R2}}=-\frac{D}{m_{l}^{\alpha}C_{l}^{\alpha,T_{R2}}\left(1-k_{\alpha}\right)L_{Rb}}\left[\Gamma\left[\left(\frac{1}{R_{root}}-\frac{1}{R_{3}}\right)-\frac{1}{R_{2}}-\frac{1}{R}\right]-GL_{Rb}\right]$ (S32b)

where $C_{l}^{\alpha,T_{R1}}$and $C_{l}^{\alpha,T_{R2}}$are the solute concentrations of liquid at the α/liquid interface with temperatures T_R1_ and T_R2_, respectively. For this condition, the TGZM effect restricts the solidification of the tip of dendrite arm at the back edge induced by the Gibbs-Thomson effect. By referring to assumptions 2–3, the relation between$C_{l}^{\alpha,T_{R1}}$and $C_{l}^{\alpha,T_{R2}}$is:

$C_{l}^{\alpha,T_{R1}}-C_{l}^{\alpha,T_{R2}}=\frac{G}{m_{l}^{\alpha}}\cdot\left(2R_{2}\right)$ (S33)

$\left.\frac{dR_{2}}{dt}\right|_{stageI}=\frac{1}{2}\left(\left.\frac{dR_{2}}{dt}\right|_{T_{R1}}+\left.\frac{dR_{2}}{dt}\right|_{T_{R2}}\right)$ (S34)

Comparison between Equation S32a and S32b shows that the dissolution rate at T_R2_ is smaller than the solidification rate at T_R1_, thus, the radius of the tip of secondary dendrite arm gradually increases as directional solidification proceeds.

**2.2 Stage II**

When peritectic reaction occurs below T_P_, β phase forms and quickly envelopes the dendrite arm of primary α phase. As shown in **Figure S6**, β phase which forms on the front/back edge of the root of secondary dendrite arm of α phase, is located at T_R1_/T_R2_ (T_R1_>T_R2_). In this case, as shown in Figure S6, between the liquid layer with temperatures ranging from T_1_′ to T_2_′, the solute concentration difference by the TGZM effect is larger than that by the Gibbs-Thomson effect. Thus, similar to what has been discussed in stage I, the TGZM effect restricts the dissolution by the Gibbs-Thomson effect which occurs at the front edge of the root of secondary dendrite arm. Therefore, the solidification of peritectic β phase induced by the TGZM effect occurs at the front edge of the tip of secondary dendrite arm. Besides, the TGZM effect accelerates the solidification by the Gibbs-Thomson effect which occurs at the front edge of the tip of secondary dendrite arm. As a result, β phase should solidify at T_R1_ and dissolve at T_R2_. The relevant part of the phase diagram of a binary peritectic system below T_P_ is illustrated in Figure S6, the distribution of solute concentration in phases is also presented. Similar to what has been proposed in stage I, at T_R1_:

$\left.\frac{dR_{2}}{dt}\right|_{T_{R1}}=-\frac{D}{m_{l}^{\beta}C_{l}^{\beta,T_{R1}}\left(1-k_{\beta}\right)L_{Ra}}\left[\Gamma\left[\left(\frac{1}{R_{root}}-\frac{1}{R_{3}}\right)-\frac{1}{R_{2}}-\frac{1}{R}\right]+GL_{Ra}\right]$ (S35a)

The dissolution rate of β phase at T_R2_ in stage II can be given by:

$\left.\frac{dR_{2}}{dt}\right|_{T_{R2}}=-\frac{D}{m_{l}^{\beta}C_{l}^{\beta,T_{R2}}\left(1-k_{\beta}\right)L_{Rb}}\left[\Gamma\left[\left(\frac{1}{R_{root}}-\frac{1}{R_{3}}\right)-\frac{1}{R_{2}}-\frac{1}{R}\right]-GL_{Rb}\right]$ (S35b)

where $m_{l}^{\beta}$ is the liquidus slope of peritectic β phase, $C_{l}^{\beta,T_{R1}}$and $C_{l}^{\beta,T_{R2}}$are the solute concentrations of liquid at the β/liquid interface at T_R1_ and T_R2_, respectively. The relation between $C_{l}^{\beta,T_{R1}}$and $C_{l}^{\beta,T_{R2}}$is:

$C_{l}^{\beta,T_{R1}}-C_{l}^{\beta,T_{R2}}=\frac{G}{m_{l}^{\beta}}\cdot\left(2R_{2}\right)$ (S36)

$\left.\frac{dR_{2}}{dt}\right|_{stageII}=\frac{1}{2}\left(\left.\frac{dR_{2}}{dt}\right|_{T_{R1}}+\left.\frac{dR_{2}}{dt}\right|_{T_{R2}}\right)$ (S37)

Similar to what has been discussed at the root of secondary dendrite arms, it can be concluded that the peritectic reaction rate is smaller than the β phase dissolution rate by the coupling effect of the TGZM and Gibbs-Thomson effects, and this difference increases as solidification proceeds. In this case, the rate of the peritectic reaction is not taken into account in stage II. As the dissolution rate at T_R2_ is larger than the solidification rate at T_R1_, β phase previously forms at T_R2_ dissolves completely first at temperature T_Q_, then stage III initiates.

**2.3 Stage III**

As has been discussed in Section 4.2 of the manuscript, if the β phase at the back edge of the root of arm dissolves completely first, both α and β phases are involved in the detachment process. As shown in **Figure S7**, the oblique solid straight lines above/below T_P_ are the liquidus lines of primary/peritectic phases. The oblique dashed straight lines below T_P_ are the extended liquidus lines of primary phase. After peritectic reaction, the thickness of peritectic phase enclosing the tip of the tear-shaped secondary dendrite arm is larger than that enclosing the root of the secondary dendrite arm. Thus, at the initial of stage III, β phase dissolves at the back side of the tip of the secondary dendrite arm; and dissolution of α phase at the back side of the tip will take place after complete dissolution of β phase enclosing it.

In this case, as shown in Figure S7a, within the liquid layer ranging from T_1_′ to T_R1_, the TGZM effect is important according to the discussion in Section 1.1. For the liquid layer ranging from T_R2_ to T_2_′, as shown in Figure 7a, the TGZM effect is also important. Thus β phase should solidify at T_R1_ and α phase should dissolve at T_R2_.

Thus, for the front edge, similar to what has been proposed in stage II, one has:

$\left.\frac{dR_{2}}{dt}\right|_{T_{R1}}=-\frac{D}{m_{l}^{\beta}C_{l}^{\beta,T_{R1}}\left(1-k_{\beta}\right)L_{Ra}}\left[\Gamma\left[\left(\frac{1}{R_{root}}-\frac{1}{R_{3}}\right)-\frac{1}{R_{2}}-\frac{1}{R}\right]+GL_{Ra}\right]$ (S38)

where $C_{l}^{\beta,T_{R1}}$is the solute concentration of liquid at the β/liquid interface at T_R1_. For the back edge of root of dendrite arm, one has:

$T_{2}^{'}=T_{\beta}+m_{l}^{\beta}C_{l}^{\beta,T_{2}^{'}}$ (S39a)

$T_{R2}=T_{\alpha}+m_{l}^{\alpha}C_{l}^{\alpha,T_{R2}}+\Gamma\left[\left(\frac{1}{R_{root}}-\frac{1}{R_{3}}\right)-\frac{1}{R_{2}}-\frac{1}{R}\right]$ (S39b)

Simultaneously, due to assumptions 2 and 3, concentration gradient resulting from the temperature gradient is established within the interdendritic liquid layer:

$T_{R2}=T_{2}^{'}+GL_{Rb}$ (S39c)

Combining the above three equations:

$C_{l}^{\beta,T_{2}^{'}}-C_{l}^{\alpha,T_{R2}}=\frac{1}{m_{l}^{\beta}}\left[\left(m_{l}^{\beta}-m_{l}^{\alpha}\right)\left(C_{LP}-C_{l}^{\alpha,T_{R2}}\right)+\Gamma\left[\left(\frac{1}{R_{root}}-\frac{1}{R_{3}}\right)-\frac{1}{R_{2}}-\frac{1}{R}\right]-GL_{Rb}\right]$ (S40)

A flux is induced by the composition gradient across the liquid with temperatures ranging from T_R2_ to T_2_′:

$J=D\frac{C_{l}^{\beta,T_{2}^{'}}-C_{l}^{\alpha,T_{R2}}}{L_{Rb}}$ (S41)

By applying a mass balance for a small displacement *dR_2_* of the α/liquid interface,

$J=D\frac{C_{l}^{\beta,T_{2}^{'}}-C_{l}^{\alpha,T_{R2}}}{L_{Rb}}=\frac{dR_{2}}{dt}\left(C_{l}^{\alpha,T_{R2}}-C_{\alpha}^{l,T_{R2}}\right)$ (S42)

Combining the above equations, the dissolution rate of α phase at T_R2_ is:

$\left.\frac{dR_{2}}{dt}\right|_{T_{R2}}=\frac{D}{m_{l}^{\beta}C_{l}^{\alpha,T_{R2}}L_{Rb}\left(k_{\alpha}-1\right)}\left[\Gamma\left(\frac{1}{R_{root}}-\frac{1}{R_{3}}-\frac{1}{R_{2}}-\frac{1}{R}\right)-GL_{Rb}+\left(m_{l}^{\beta}-m_{l}^{\alpha}\right)\left(C_{LP}-C_{l}^{\alpha,T_{R2}}\right)\right]$ (S43)

where $C_{l}^{\alpha,T_{R2}}$is the solute concentration of liquid at the α/liquid interface at T_R2_. The relation between $C_{l}^{\beta,T_{R1}}$in Equation S10 and $C_{l}^{\alpha,T_{R2}}$is:

$\begin{matrix} C_{l}^{\alpha,T_{R2}}-C_{l}^{\beta,T_{R1}}=\frac{T_{P}-T_{R1}}{m_{l}^{\beta}}-\frac{T_{P}-T_{R2}}{m_{l}^{\alpha}} \\ =\left(T_{P}-T_{R1}\right)\left(\frac{1}{m_{l}^{\beta}}-\frac{1}{m_{l}^{\alpha}}\right)-\frac{G}{m_{l}^{\alpha}}\cdot\left(2R_{2}\right) \end{matrix}$ (S44)

It can be observed from Equation S43 that the dissolution rate increases with increasing $C_{l}^{\alpha,T_{2}}$. According to Equation S44, $C_{l}^{\alpha,T_{2}}$increases with increasing temperature gradient *G* but decreases with the decrease of temperature at the β/liquid interface T_R1_. Therefore, the dissolution rate of α phase can be significantly accelerated by increasing the temperature gradient.

$\left.\frac{dR_{2}}{dt}\right|_{stageIII}=\frac{1}{2}\left(\left.\frac{dR_{2}}{dt}\right|_{T_{R1}}+\left.\frac{dR_{2}}{dt}\right|_{T_{R2}}\right)$ (S45)

**3. Variation of radius R_3_ during detachment**

During the detachment of secondary dendrite arm, the radius *R_3_* also changes in accompany with the decrease of *R_root_*. Although Liotti et al.^2^ believed that *R_3_* increases during the detachment of secondary branches, Lu et al.^3^ has proved that both *R_root_* and *R_3_* decreased in the detachment process. The variation of *R_3_* has been illustrated in **Figure S8**, *R_3_* is the radius of the excircle (circle center is O) corresponding to *R_root_*; O' is the circle center of the excircle after *R_root_* has reduced by a distance *l*. Remelting which occurs at the root of the secondary dendrite arm is not uniform at different positions. It can be deduced from the above expressions of remelting rate at the root of secondary dendrite arm that the solid phase remelts fastest at R=*R_root_*, while the areas in the vicinity of that lag behind. Thus, the radius *R_3_* should be smaller during this detachment process. Furthermore, it can be observed from Figure S8 that the decrease of *R_3_* is much faster than that of *R_root_*. When the remelting distance is *l*, the change of the radius of excircle OO' is obviously larger than *l*. This is consistent with the measurement in NH_4_Cl-H_2_O system by Lu et al., which shows that the decrease rate of *R_3_* is about 3~4 times that of *R_root_*. It should be noted that the variation of *R_3_* with solidification time can not be directly obtained through the variation of *R_root_*(*l*). The dependence of *R_3_* on solidification time which is based on our experimental measurement can be expressed as: *R_3_= R_3_(0)-2×10^-8^t,* where *R_3_(0)* is the initial value of *R_3_*, and is obtained from the experimental results.

**4. The influence of orientation of dendrite on detachment process**

In the discussion above, it is assumed that the secondary dendrite arms are strictly perpendicular to the primary dendrite stems which grow parallel to the direction of temperature gradient. However, in fact, the experimental results show that the orientations of secondary branches more or less deviate from the direction which is strictly perpendicular to the direction of temperature gradient. As shown in Figure S1, the solute fluxes due to the curvature difference between the tip and root of the tear-shaped secondary dendrite arms transport from the tip to the root. And the directions of these fluxes are perpendicular to the solid/liquid interface of the roots. Therefore the influence of curvature difference on detachment does not depend on whether the branches are perpendicular to the direction of temperature gradient.

However, the influence of the TGZM effect on detachment is closely associated with both the magnitude and direction of temperature gradient. As shown in **Figure S9**, here the intersection angle between the solid/liquid interface of secondary branches and the direction of temperature gradient is denoted as *θ*. Then, the effective solute fluxes to the roots of secondary dendrite arms by the TGZM effect is *J_T_*·*sinθ*. It can be easily concluded that the influence of the TGZM effect on detachment is more significant when this intersection angle increases (0˂*θ*˂π/2) gradually. Besides, it can be assumed that when the TGZM effect is reduced through the change of *θ*, although the dissolution rate at the back edge of the root of secondary dendrite arm decreases, the resolidification rate at the front edge of the root of secondary dendrite arm increases simultaneously. This indicates that the influence of the orientation of secondary dendrite arm may be inconspicuous when the intersection angle *θ* is relatively small in actual directional solidification.

**Reference**

^1^Marsh, S. P. & Glicksman, M. E. Overview of geometric effects on coarsening of mushy zones. Metall. Mater. Trans. A **27A**, 557-567 (1996).

^2^Liotti, E. et al. A synchrotron X-ray radiography study of dendrite fragmentation induced by a pulsed electromagnetic field in an Al-15Cu alloy. Acta Mater. **70**, 228–239 (2014).

^3^Jackson, K. A., Hunt, J. D., Uhlmann, D. R. & Seward, T. P. On the origin of the equiaxed zone in castings. Trans. AIME **236**, 149-158 (1966).

^4^Peng, P. et al. Effect of peritectic reaction on dendrite coarsening in directionally solidified Sn-36at.%Ni alloy. J. Mater. Sci. **47**, 6108-6117 (2012).

^5^Liu, D. M. et al. Secondary dendrite arm migration caused by temperature gradient zone melting during peritectic solidification. Acta Mater. **60**, 2679-2688 (2012).

^6^Vandyoussefi, M., Kerr, H. W. & Kurz, W. Two-phase growth in peritectic Fe–Ni alloys. Acta Mater. **48**, 2297-2306 (2000).

^7^Wu, Y., Piccone, T. J., Shiohara, Y. & Flemings, M. C. Dendritic growth of undercooled nickel-tin: Part II. Metall. Trans. A **18A**, 925-932 (1987).

^8^Sumida, M. Evolution of two phase microstructure in peritectic Fe–Ni alloy. J. Alloys Compd. **349**, 302-310 (2003).


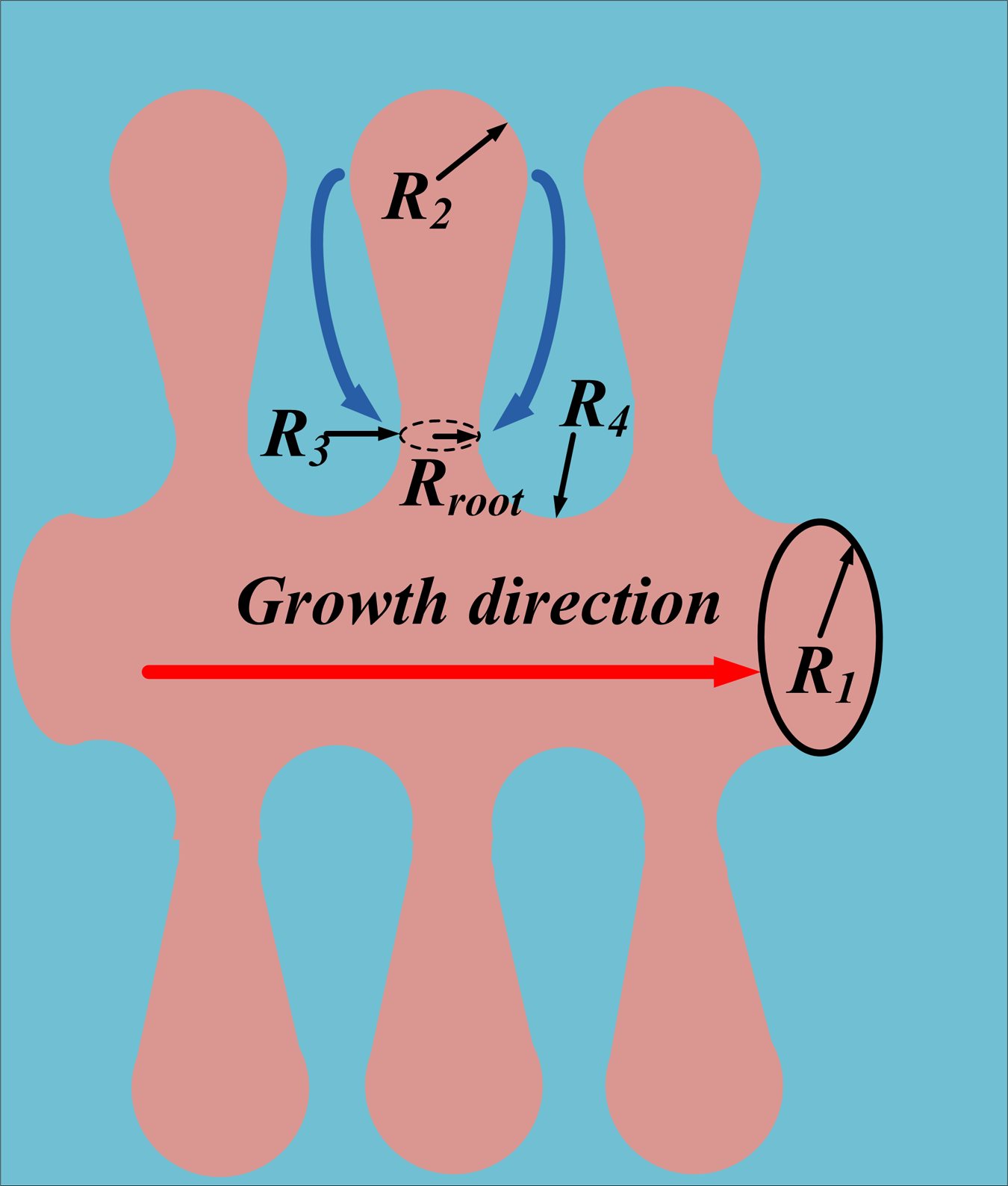


**Figure S1** Schematic illustration of different radii at the dendritic structure which is composed of primary dendrite stem and secondary dendrite branch.


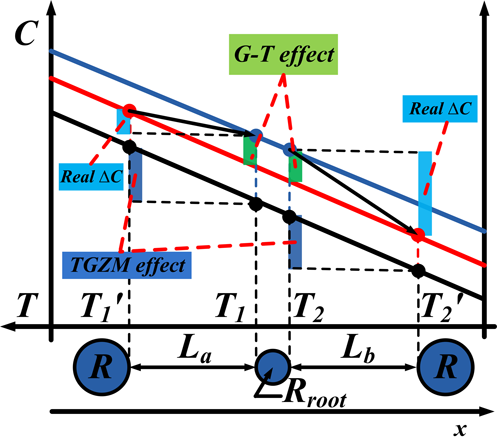


**Figure S2** Schematic illustration of stage I of the detachment process in the root of the secondary dendrite arm: the relevant part of the phase diagram of a binary peritectic system above T_P_ and the solute concentration distribution in the interdendritic liquid.


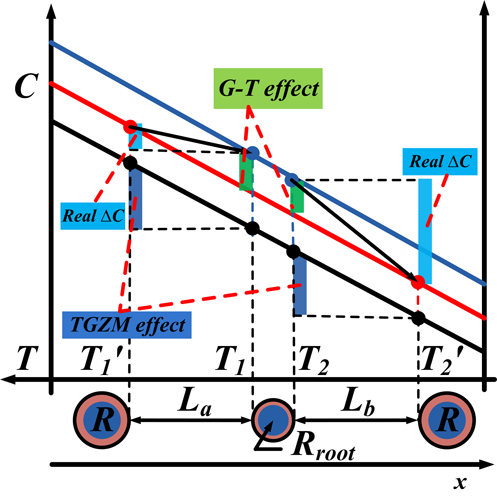


**Figure S3** Schematic illustration of stage II of the detachment process in the root of the secondary dendrite arm: the relevant part of the phase diagram of a binary peritectic system below T_P_ and the solute concentration distribution in the interdendritic liquid.


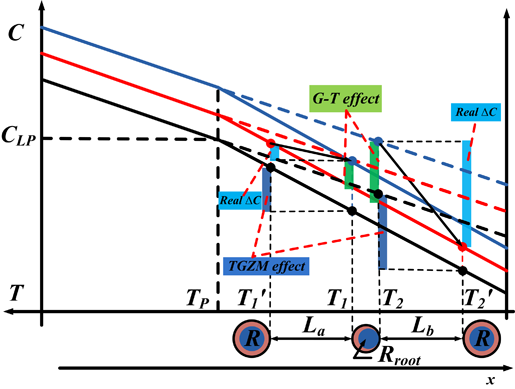


**(a)**


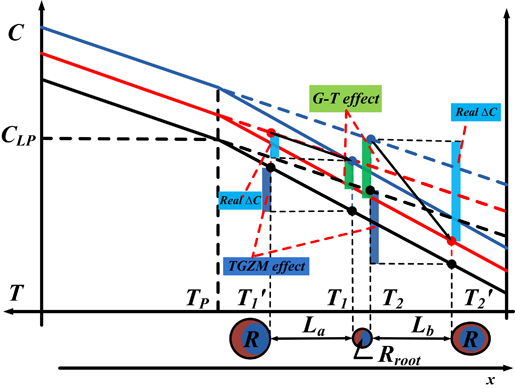


**(b)**


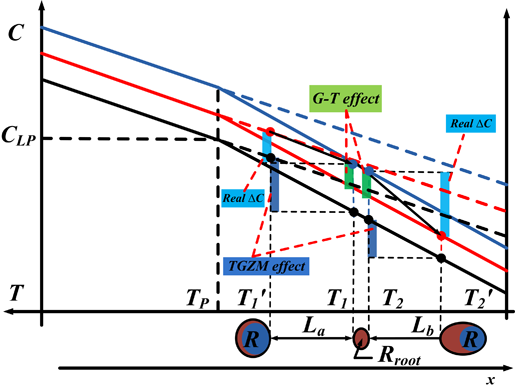


**(c)**


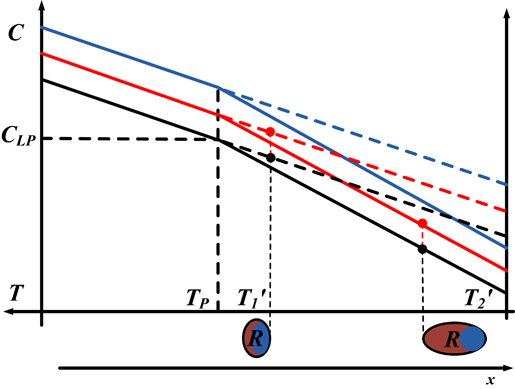


**(d)**

**Figure S4** Schematic illustration of stage III of the detachment process in the root of the secondary dendrite arm: (a) The initial of stage III, (b) complete dissolution of β phase at T_1_', (c) the thinner secondary dendrite arm is of peritectic β, (d) complete dissolution of the thinner secondary dendrite arm.


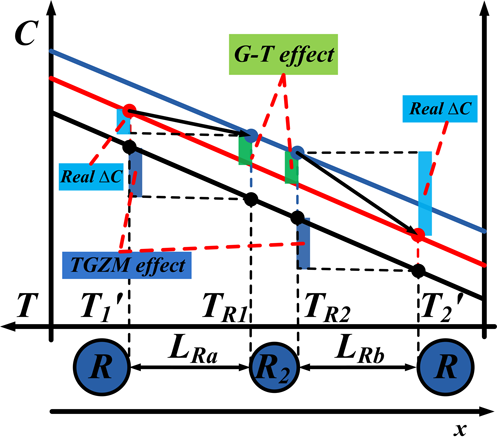


**Figure S5** Schematic illustration of stage I of the detachment process in the tip of the secondary dendrite arm: the relevant part of the phase diagram of a binary peritectic system above T_P_ and the solute concentration distribution in the interdendritic liquid.


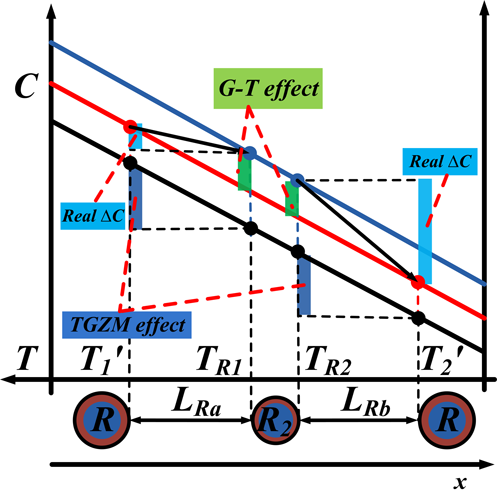


**Figure S6** Schematic illustration of stage II of the detachment process in the tip of the secondary dendrite arm: the relevant part of the phase diagram of a binary peritectic system below T_P_ and the solute concentration distribution in the interdendritic liquid.


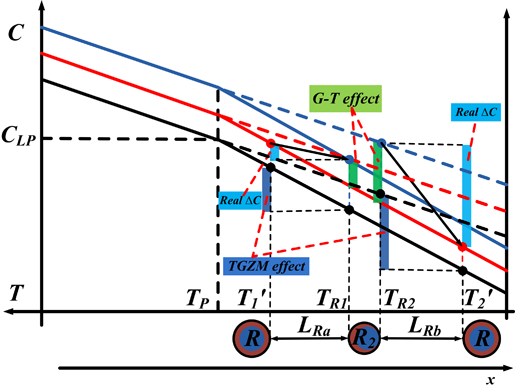


**(a)**


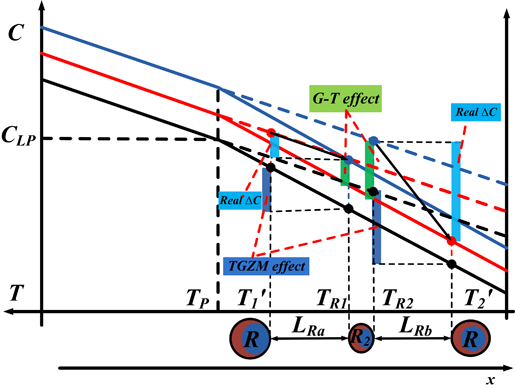


**(b)**


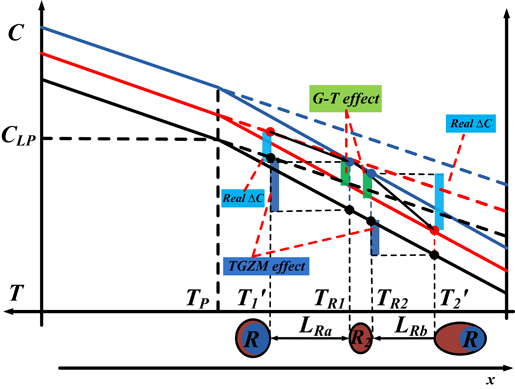


**(c)**


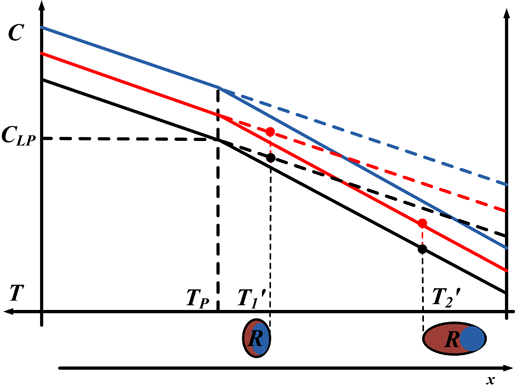


**(d)**

**Figure S7** Schematic illustration of stage III of the detachment process in the tip of the secondary dendrite arm: (a) The initial of stage III, (b) complete dissolution of β phase at T_1_', (c) the thinner secondary dendrite arm is of peritectic β, (d) complete dissolution of the thinner secondary dendrite arm.


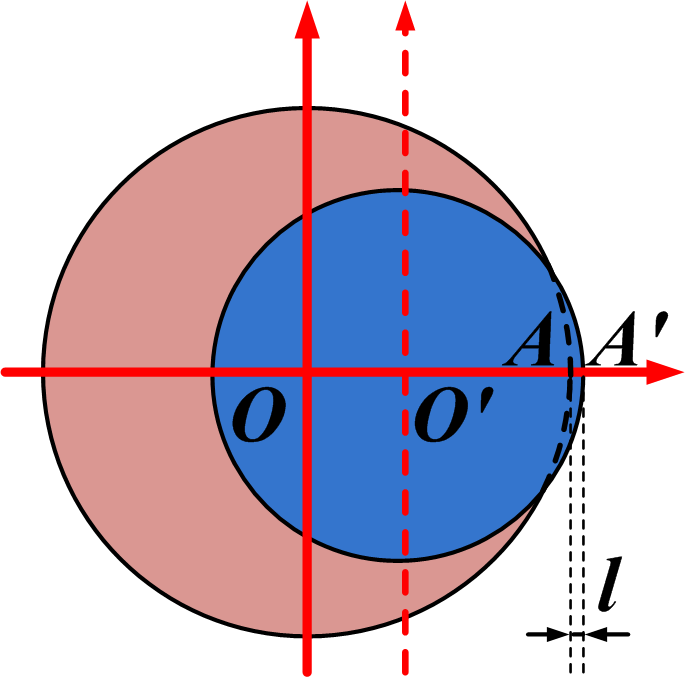


**Figure S8** Schematic illustration of variation of the radius R_3_ during the detachment process.


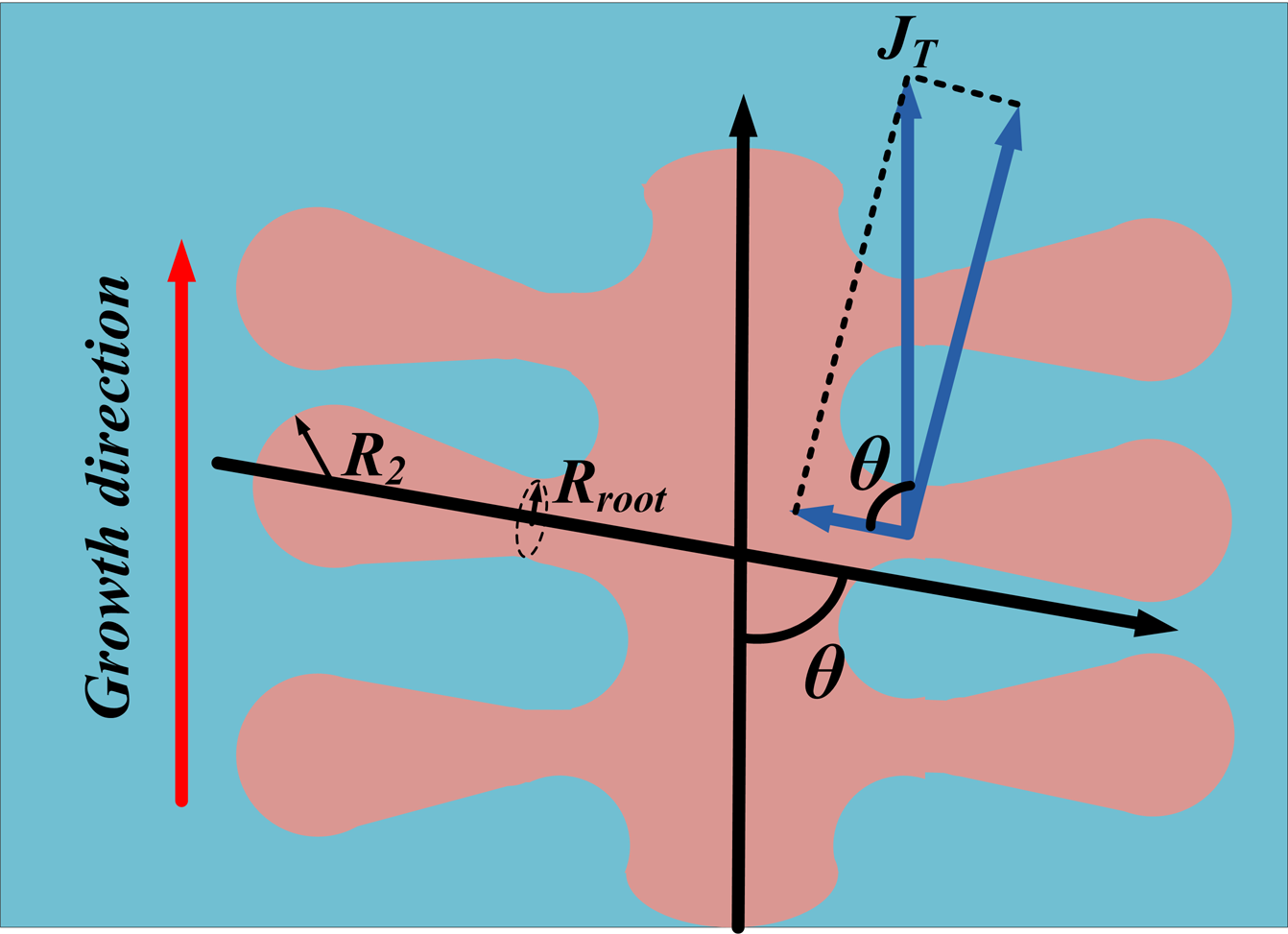


**Figure S9** Illustration of the dependence of the influence of the TGZM effect on detachment on intersection angle *θ*.

1. ^^ [↑](#footnote-ref-2)
